# Enhancing RNA-seq analysis by addressing all co-existing biases using a self-benchmarking approach with 2D structural insights

**DOI:** 10.1093/bib/bbae532

**Published:** 2024-10-19

**Authors:** Qiang Su, Yi Long, Deming Gou, Junmin Quan, Qizhou Lian

**Affiliations:** Faculty of Synthetic Biology, Shenzhen University of Advanced Technology, Shenzhen Key Laboratory of Quantitative Synthetic Biology, Shenzhen Institute of Synthetic Biology, Shenzhen Institutes of Advanced Technology, Chinese Academy of Sciences, 1068 Xueyuan Avenue, Nanshan District, Shenzhen, 518055, China; State Key Laboratory of Chemical Oncogenomics, School of Chemical Biology and Biotechnology, Peking University Shenzhen Graduate School, 2199 Lishui Avenue, Nanshan District, Shenzhen, 518055, China; Institute of Chemical Biology, Shenzhen Bay Laboratory, Gaoke International Innovation Center A14, Guangqiao Road, Guangming District, Shenzhen, 518132, China; Shenzhen Key Laboratory of Microbial Genetic Engineering, Vascular Disease Research Center, College of Life Sciences and Oceanography, Shenzhen University, 1066 Xueyuan Street, Nanshan District, Shenzhen, 518055, China; State Key Laboratory of Chemical Oncogenomics, School of Chemical Biology and Biotechnology, Peking University Shenzhen Graduate School, 2199 Lishui Avenue, Nanshan District, Shenzhen, 518055, China; Faculty of Synthetic Biology, Shenzhen University of Advanced Technology, Shenzhen Key Laboratory of Quantitative Synthetic Biology, Shenzhen Institute of Synthetic Biology, Shenzhen Institutes of Advanced Technology, Chinese Academy of Sciences, 1068 Xueyuan Avenue, Nanshan District, Shenzhen, 518055, China; Cord Blood Bank, Guangzhou Institute of Eugenics and Perinatology, Guangzhou Women and Children’s Medical Center, Guangzhou Medical University, 9 Jinshui Road, Tianhe District, Guangzhou, 510623, China; State Key Laboratory of Pharmaceutical Biotechnology, and Department of Medicine, The University of Hong Kong, 102 Pok Fu Lam Road, Hong Kong SAR, China

**Keywords:** MFE-GSB, k-mer, Gaussian distribution, RNA-seq

## Abstract

We introduce a groundbreaking approach: the minimum free energy–based Gaussian Self-Benchmarking (MFE-GSB) framework, designed to combat the myriad of biases inherent in RNA-seq data. Central to our methodology is the MFE concept, facilitating the adoption of a Gaussian distribution model tailored to effectively mitigate all co-existing biases within a *k*-mer counting scheme. The MFE-GSB framework operates on a sophisticated dual-model system, juxtaposing modeling data of uniform *k*-mer distribution against the real, observed sequencing data characterized by nonuniform *k*-mer distributions. The framework applies a Gaussian function, guided by the predetermined parameters—mean and SD—derived from modeling data, to fit unknown sequencing data. This dual comparison allows for the accurate prediction of *k*-mer abundances across MFE categories, enabling simultaneous correction of biases at the single *k*-mer level. Through validation with both engineered RNA constructs and human tissue RNA samples, its wide-ranging efficacy and applicability are demonstrated.

## Introduction

RNA sequencing (RNA-seq) stands as a cornerstone technology for in-depth analysis of the transcriptome, providing unparalleled insights into tissue-specific transcription, patterns associated with diseases, and unique cellular signatures [1, 2]. This powerful approach employs both short-read and long-read sequencing technologies. Notably, long-read sequencing is acclaimed for its capability to sequence entire transcripts in a single effort, thereby significantly enriching our understanding of transcript diversity and intricacies [3]. Long-read sequencing, however, confronts certain challenges, including lower throughput and increased error rates. These issues may impact the accuracy of detecting gene expressions across diverse levels of expression [4, 5]. In scenarios where comprehensive genomic exploration is the objective, short-read sequencing stands out as the method of choice [6]. This approach excels by producing a vast number of short reads that together ensure an exhaustive capture of the transcriptome [7, 8], encompassing even transcripts of low abundance, and yielding data of exceptional quality that is crucial for sophisticated analyses [9]. The effectiveness of short-read RNA-seq is, however, contingent upon a carefully designed workflow. This workflow is essential for methodically fragmenting long, complex transcripts into shorter, more manageable sequences [8, 10]. Despite the strengths of RNA-seq protocols, they are susceptible to introducing biases that may result in irregular distribution of reads across transcripts. Such biases can compromise the reconstruction and quantification of transcripts [11–13], affecting the overall accuracy of the study.

Navigating the intricate web of biases present in short-read RNA-seq datasets represents a formidable challenge. This spectrum of biases encompasses, among others, GC bias, where sequencing coverage is influenced by guanine–cytosine (GC) content; fragmentation or degradation bias, related to the differential stability of RNA fragments; library preparation bias, stemming from preferences in hexamer priming and Polymerase Chain Reaction (PCR) amplification efficiencies; mapping bias, linked to the intrinsic characteristics of RNA molecules; and experimental technical bias, encompassing variations due to differences in sequencing depth and batch effects [14–20]. Conventional strategies to mitigate these biases typically involve creating specific correction factors by comparing observed sequencing data against a benchmark reference [17, 21–24]. While such approaches can diminish the impact of certain biases on the distribution of sequencing reads, achieving a completely unbiased sequencing outcome is challenging for several reasons. Firstly, most existing bias mitigation strategies tackle biases in isolation, failing to account for their concurrent occurrence and interplay. Secondly, the complex interrelationship among these biases complicates the task of isolating and correcting them individually. Furthermore, current methods for bias correction rely heavily on empirical sequencing data, which are themselves affected by these varied biases. The existence of unknown biases in sequencing data further complicates the situation, as there is presently no comprehensive methodology capable of adapting to these unidentified factors in RNA-seq data [25]. These challenges highlight the critical need for a novel, more sophisticated model that can address multiple biases simultaneously in a cohesive manner, without depending on potentially biased empirical data. Such a model would represent a significant advancement in the field, offering the possibility of more reliable and accurate RNA-seq data analysis.

Our research presents a novel Gaussian Self-Benchmarking (GSB) framework, which is rooted in the concept of minimum free energy (MFE). This approach utilizes a Gaussian distribution model to accurately identify and correct all co-occurring biases in a *k*-mer counting framework. The foundation of our method is the observed Gaussian distribution of guanine (G) and cytosine (C) across natural transcripts when *k*-mer counts are categorized based on their GC content. By exploiting the correlation between 1D GC content and 2D MFE structural attributes, we transform the GC-content-based distribution into an MFE-focused distribution that conforms to Gaussian distribution principles. This transformation allows us to establish the MFE-GSB framework effectively. To address the concurrent biases, the MFE-GSB framework requires dual models. One model operates under a theoretical framework with a uniform distribution of *k*-mers across the transcript. Conversely, the second model emerges from the empirical sequencing data, reflecting the actual, uneven distribution of *k*-mers along the transcript. The process begins by sorting *k*-mers into specific MFE bins for uniformly distributed modeling data. These *k*-mers are then aggregated by their MFE, and these aggregates are modeled using a Gaussian distribution focused on determining each distribution’s mean and SD, reflecting each transcript’s unique characteristics. Similarly, sequencing data are categorized and aggregated according to their MFE content. The framework applies a Gaussian function, guided by the predetermined parameters, to fit the sequencing *k*-mer count aggregates across MFE categories. Predictions made by this Gaussian function fitting act as an unbiased indication for abundance within each MFE category. These predictions, once averaged across each MFE category, play a crucial role in systematically reducing bias at specific *k*-mers within the transcript. Since MFE delineates RNA’s 2D structural properties—which are inherently more complex than 1D structures—the MFE-GSB framework provides a sophisticated mechanism for high-resolution bias identification and correction. A unique feature of our GSB-MFE framework is its self-benchmarking system, which establishes a theoretical benchmark for the adjustment of biases, steering clear of the bias-specific approximations characteristic of traditional empirical methods. These parameters are exclusively derived from theoretical modeling, independent from any empirical sequencing data. This approach allows for more accurate predictions of abundance levels by systematically eliminating bias through parameter-fixed fitting. The validity of MFE-GSB has been rigorously tested through a comprehensive validation protocol that combines theoretical frameworks with empirical evaluations. This includes experiments with both artificial RNA constructs and authentic human tissue RNA samples, affirming the robustness and wide applicability of our approach.

## Results

**Fig. 1:**
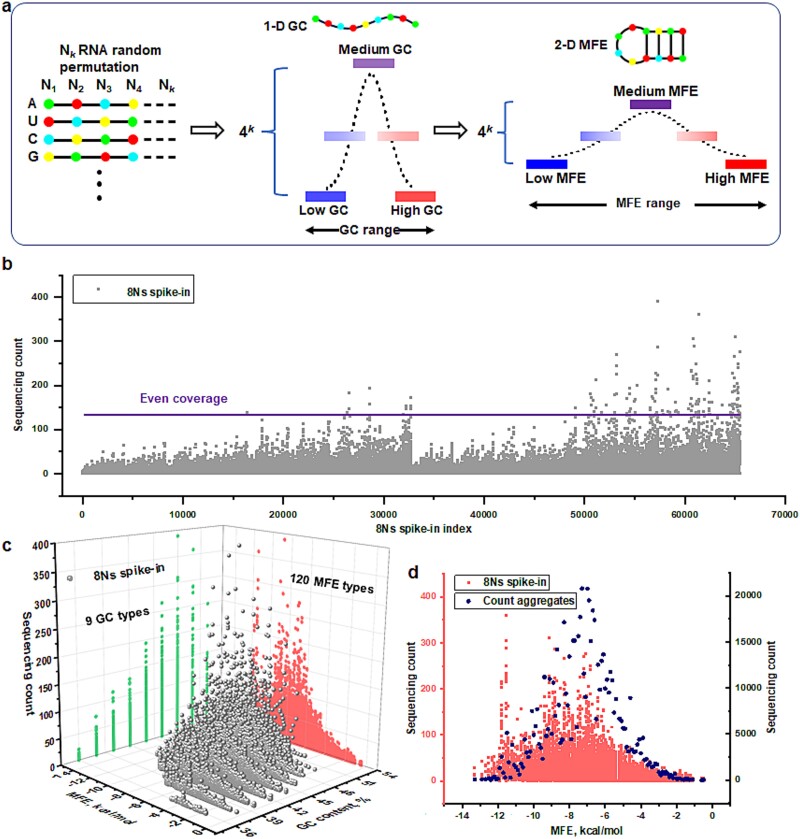
Assessing and enhancing the distribution of sequencing counts through MFE analysis. (a) The MFEGSB method analyzes random G and C base arrangements in *k*-mer sequences, using the binomial distribution to systematically study *k*-mers with identical MFEs. It proposes a strategy for classifying *k*-mers by their MFE and aggregating their counts within specified MFE groups. This approach highlights a Gaussian distribution of *k*-mer counts, revealing the structured distribution of *k*-mers based on their energy profiles. (b) The sequencing count profile evaluates distribution variability by examining 65 536 unique spike-in RNA sequences with distinct nucleotide patterns (4^8 variation). This approach efficiently maps out distribution differences across a wide spectrum of sequences. (c) The 3D sequencing count plot visualizes the distribution of sequencing counts across 65 536 unique spike-in RNA templates, each identified by its GC content and MFE. The sequencing data are categorized and centralized based on these two parameters, GC content and MFE. (d) Aggregating sequencing counts of spike-in RNA sequences by their matching MFE values reveals the development of bell-shaped distributions among different MFE categories.

The theoretical foundation and empirical evidence supporting the MFE-GSB algorithm are based on employing the binomial distribution model of GC content as a statistical framework (as shown in supporting information, Theoretical Consideration section). To investigate the relationship between the MFE of an RNA sequence and its distribution in RNA-sequencing (RNA-seq) data, we conducted an experiment using synthesized, fixed-length spike-in RNA sequences. The premise of our study is rooted in the foundational principle that an RNA strand comprising (k) nucleotides can theoretically adopt one of (4^k) unique sequences, as nucleotides at each position can be any of the four RNA bases (as illustrated in Fig. 1a). We tailored these spike-ins to exhibit variable MFEs to specifically scrutinize how MFE influences RNA-seq read distribution. Our experimentation strategically employed circular RNA to dissect the relationship between RNA structure, MFE, and RNA-seq patterns (as provided in supporting information Fig. S1 available online at http://bib.oxfordjournals.org). The choice of circular RNA as our experimental subject underscores the significant role of MFE in influencing RNA-seq distribution patterns. In constructing our RNA templates, we specifically included a core of 50 nucleotides surrounded by 8-mer poly-A tails to minimize any potential ligation bias and ensure uniform production of circular RNA complexes. By thoroughly examining these aspects, our research sheds light on the complex dynamics between RNA sequence structure and MFE and their impact on RNA-seq results.

In the process of data handling, the use of complete spike-in sequences is a critical method for determining the read count of each RNA sequence. Each unique RNA sequence, which is represented by a sequence that contains eight variable nucleotides (N) within a 50-mer, is carefully analyzed for sequencing reads in the order of A, T, C, and G at the variable positions (as shown in Fig. 1b). A notable observation from this analysis is the significant variability in the read counts, which stands in stark contrast to the uniform distribution typically expected in theoretical modeling data. This variability in the coverage of spike-in RNAs points toward the existence of biases inherent to the RNA-seq process. To validate the complex and reproducible nature of this bias, two separate instances of spike-in-based RNA sequencing were performed, revealing consistent results with strong correlations between the replicates (as illustrated in the supporting information Fig. S2 available online at http://bib.oxfordjournals.org). This level of consistency underscores the critical influence that the natural structural attributes of each spike-in template exert on its sequencing efficiency. Recognizing this influence is vital for accurately comparing major datasets. Further analysis involved organizing the sequencing counts of spike-in RNAs according to their GC content and MFE, revealing clear stratification across nine distinct groups according to GC content, and further into 120 unique categories based on MFE (as shown in Fig. 1c). The distribution of spike-ins according to the GC or MFE index allows for a focused analysis. This organization leads to a bell-shaped distribution of sequencing counts when arranged by GC content (as illustrated in the supporting information Fig. S3 available online at http://bib.oxfordjournals.org) and MFE (as shown in Fig, 1d), clearly evident as counts from RNAs within each category are aggregated. Therefore, the distribution of sequencing counts among the spike-in RNAs, along with their associated GC content and MFE, showed significant variability (as shown in the supporting information, Fig. S4 available online at http://bib.oxfordjournals.org). However, this distribution becomes more consistent when the RNAs are organized by their GC content or MFE, underscoring the significant impact of organization by the inherent structure of RNA template. This transition highlights the pivotal role of the MFE-based organization in mitigating sequencing bias, offering insightful perspectives into the underlying mechanisms of RNA-seq.

### Advancing bias mitigation via the minimum free energy–based Gaussian Self-Benchmarking framework

**Fig. 2:**
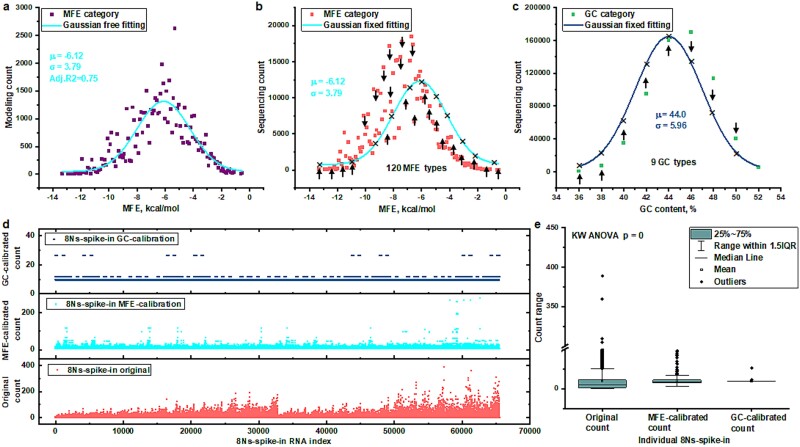
Enhancing sequencing data accuracy with MFE-GSB calibration. (a) The examination of modeling counts, classified according to their MFE, was conducted using a Gaussian distribution method. The key parameters were identified and extracted. These parameters serve as a representative measure for a targeted uniform distribution, crucial for spike-in controls. (b) The sequencing data, organized according to MFE, is then recalibrated to align with the Gaussian model, guided by the identified parameters. Discrepancies between the predicted counts and the original counts at various MFE levels are illustrated with arrows, emphasizing the differences. (c) Similarly, the sequencing data, when organized by comparing GC content with MFE, is recalibrated to conform to the Gaussian model. This process is informed by parameters predefined based on GC content. Differences between the model-predicted counts and the actual counts at various GC-content levels are visually emphasized, with arrows indicating the discrepancies. (d) For each individual spike-in, a precise level of correction is performed by comparing actual sequencing counts to those defined by the MFE-GSB and GC-based GSB benchmarks. (e) The calibration efficacy applied by MFEGSB and GC-based GSB is demonstrated via a box plot, which contrasts the sequencing counts of all spike-ins pre- and postcalibration.

The MFE-GSB framework introduces a sophisticated methodology meticulously designed to neutralize concurrent biases effectively. Central to the MFE-GSB framework is the strategic predetermination of core parameters, deliberately chosen to be independent from empirical sequencing data. This pivotal independence guarantees that the benchmarking process is not compromised by the variabilities and inherent biases that are typically present in empirical data. The methodology initiates with the construction of a theoretical model for spike-in RNA data, predicated on the assumption of even coverage across various spike-in RNA templates. The model’s counts are then systematically categorized and compiled, followed by a refinement phase utilizing a mathematical fitting procedure that employs the Gaussian distribution function (as referenced in Fig. 2a). This phase is carried out with exacting precision, ensuring that the mean and SD parameters precisely encapsulate the intricacies of the synthetic spike-in RNA components. After defining the theoretical parameters, the sequencing data—systematically organized and compiled by MFE—is refined using a predefined Gaussian distribution marked by transcript-specific mean and SD values (as depicted in Fig. 2b). Introducing fixed parameters at this stage ensures a precise and unbiased representation of counts for each category under MFE evaluation. Any discrepancies observed between these predicted count and the actual sequencing data are then meticulously corrected through a calibration endeavor, aiming to comprehensively eliminate any and all potential biases. To facilitate a comparison with the MFE-GSB approach, the GC-based SGB framework is also applied to the same sequencing data, which is stratified according to GC content (as illustrated in Fig. 2c). The data are categorized into 120 groups according to MFE for the MFE-GSB approach, in contrast to the simplification into 9 groups based on GC content for the SGB framework. Although adjustments can be made to bridge the gap between the predicted counts derived from GC content and the actual counts, the coarse granularity provided by the 9 levels of GC content significantly lags behind the detailed 120 MFE levels utilized for sequencing data categorization. Consequently, the MFE-based calibration approach is expected to offer a more accurate correction of bias, thanks to its finer granularity in classification.

This technique leverages the principles of Gaussian distribution to analyze count data, resulting in the generation of unbiased estimators within sequence analyses across different categories, such as MFE or GC content. By calculating the average predicted counts for all related *k*-mers within each category, it markedly reduces the biases commonly associated with individual spike-ins (as illustrated in Fig. 2d). This method ensures precise count calibration for each RNA spike-in, allowing for a straightforward comparison between actual sequence counts and theoretical predictions based on either GC content or MFE for each RNA spike-in template. The side-by-side comparison of the original sequencing data’s count distributions with the predicted counts (as shown in Fig. 2e) highlights the complex biases introduced during sequencing. It also emphasizes the effectiveness of this Gaussian-based Smoothing (GSB) framework in mitigating such biases. While both MFE predictions and GC content analysis play crucial roles in calibrating sequencing *k*-mer counts, the GSB method especially underscores the importance of MFE. This approach offers a more detailed, precise, and insightful examination of *k*-mer count distributions, surpassing the more simplistic models that only consider GC content. By employing predefined parameters to systematically address bias, this advanced approach sets a new standard in RNA sequencing analysis. It represents a significant leap forward in achieving truly unbiased quantification and greatly enhances the interpretation of RNA sequencing data.

### Assessing the efficacy of the minimum free energy–based Gaussian Self-Benchmarking model in mitigating bias within natural transcript

**Fig. 3:**
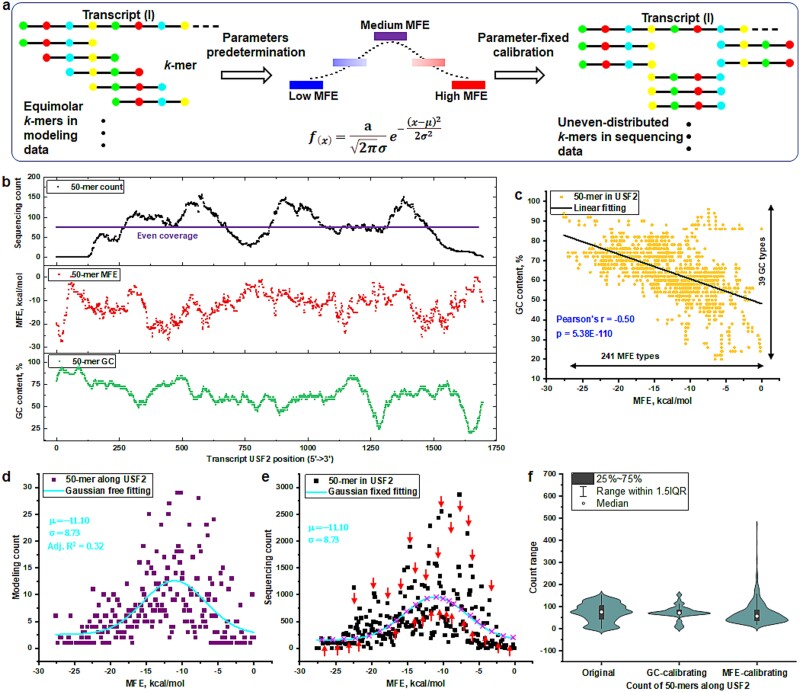
Overview of an MFE-GSB approach for adjusting natural transcript bias in sequencing data analysis. (a) The MFE-GSB method refines *k*-mer modeling counts from natural transcripts by sorting them via MFEs for fitting into a Gaussian model. This approach sets core parameters to accurately adjust 50-mer sequencing counts, revealing inherent biases through observed discrepancies between predicted and actual counts. (b) The examination of sequencing counts, GC content, and MFE values for 50-mer starting from the 5′ end of the human USF2–201 transcript (ENST00000222305). Additionally, the model displays the distribution of the 50-mer with an assumption of even distribution. (c) The linear regression analysis conducted on 50-mer sequences from USF2 reveals an inverse linear relationship between the 39 identified types of GC content and the 241 variants of the MFE. (d) The aggregate counts from 241 unique MFE drawn from the even 50-mer distribution data have been analyzed through fitting with a Gaussian distribution function. This analysis yields outcomes including the mean, SD, and the coefficient of determination (R^2). (e) The sequencing counts of 50-mer sequences, categorized by their MFE values in the actual sequencing data, were aligned to a Gaussian distribution function defined by the parameters set forth in (d). This process entailed applying the required calibration changes to each MFE category, as denoted by the inclusion of directional arrows. (f) A comparative analysis of the individual count distribution illustrates the original versus GC-content and MFE-based calibrated sequencing counts for individual 50-mers across the transcript.

To enhance the credibility of the MFE-GSB framework, it is crucial to thoroughly assess its accuracy and reliability. This assessment involves utilizing actual sequencing data derived from a genuine transcriptome. Our methodology entails segmenting a complete human transcriptome sequence, beginning from its 5′ end, into specified lengths termed as *k*-mers (as illustrated in Fig. 3a). For each *k*-mer, which is evenly distributed along the transcript, we compute the MFE and then categorize each based on their MFE values. The MFE-GSB framework subsequently utilizes a Gaussian distribution function to model the counts. This critical step helps identify essential parameters, such as the mean and SD. With these parameters, we then apply the Gaussian distribution function to the sequencing counts organized by MFE. This process is fundamental for calibrating the sequencing counts within each MFE category through predictive adjustments offered by the GSB framework. Furthermore, this approach goes beyond merely adjusting counts at the MFE-category level; it also normalizes the counts at the *k*-mer level. This is achieved by calculating the average of the adjusted prediction counts for each MFE category. These averages are then distributed among individual *k*-mers within that category. By employing this technique, we standardize the representation of *k*-mer counts across different MFE categories. This standardization is vital as it ensures that the distribution of *k*-mers within the respective categories is considered uniformly.

Our study centers on an in-depth examination of the USF2-201 transcript template, noted for its median transcript length and noticeable abundance. We employ a stacked plot to simultaneously depict the relationship between 50-mer sequencing counts, GC content, and MFE, utilizing sequencing data sourced from HEK293T cells (as referred to Fig. 3b). This analysis underscores the significant disparities in GC content, MFE, and sequencing counts among 50-mers spanning natural transcripts, in contrast to the even distribution observed within modeled data. In the development of the MFE-GSB model, the Gaussian distribution function that leverages MFE is adapted from an existing function considering GC content. This adaptation underscores a significant link between the GC content and the MFE values of *k*-mers. To illustrate this relationship, a regression analysis focusing on the GC content and MFE within USF2 data (as shown in Fig. 3c) was performed. The analysis resulted in a closely fitting linear regression, providing a strong foundational justification for the creation of the MFE-GSB model. Similar tightly fitting linear regression models were also observed in analyses of other transcript-based datasets, such as ACTB, EMP1, and PGK1, among others (as illustrated in the supporting information Fig. S5 available online at http://bib.oxfordjournals.org). This consistent linear association between GC content and MFE across a range of natural transcripts significantly enhances our comprehension of the linkage between these two critical variables. The investigation included a total of 1697 *k*-mers across USF2, which were classified into 39 distinct groups based on GC content and 241 groups based on MFE. The discovery of similar structural patterns in other transcripts reveals a wider variety of MFE distinctions compared to GC content. Such detailed categorization suggests that MFE can more precisely describe the structural nuances of *k*-mers than GC content alone.

The aggregate counts obtained from 241 unique MFE categories, which are sourced from a uniform distribution of 50-mer sequences, have undergone a comprehensive examination. This examination involves the application of a Gaussian distribution function to fit the MFE-categorized data. Through this sophisticated analytical approach, several vital statistical metrics were determined, such as the mean, which indicates the central tendency of the MFE dataset, and the SD, which sheds light on the dispersion or the extent to which the data points deviate from the mean MFE (as referred to Fig. 3d). This exhaustive investigation not only deepens the understanding of the MFE-specific characteristics associated with the USF2 transcript but also establishes a reference point for evaluating unknown sequencing data. The application of a Gaussian distribution function, with predetermined parameters, to model the sequencing count organized by MFE (as illustrated in Fig. 3e) revealed a noticeable discrepancy between the predicted and actual sequencing counts, indicating significant bias effects. To address these biases, adjustments for both over-sampling and under-sampling were made across all MFE categories, leading to a thorough calibration process. A comparative analysis of individual count distributions reveals variances between the original sequencing counts and the adjusted counts, which are fine-tuned for GC-content and MFE, across individual 50-mer sequences in the USF2–201 transcript (as depicted in Fig. 3f and the supporting information Fig. S6 available online at http://bib.oxfordjournals.org). The adjustment based on MFE achieves a more faithful and consistent representation of the abundance of each 50-mer compared to GC-based calibration. By correcting for these intricate biases at the single 50-mer level, the refined counts yield a dataset of enhanced accuracy. This improvement significantly bolsters the reliability of gene expression analysis and bioinformatics research.

### Evaluating Gaussian Self-Benchmarking framework and traditional smoothing methods with minimum free energy and guanine–cytosine content

**Fig. 4:**
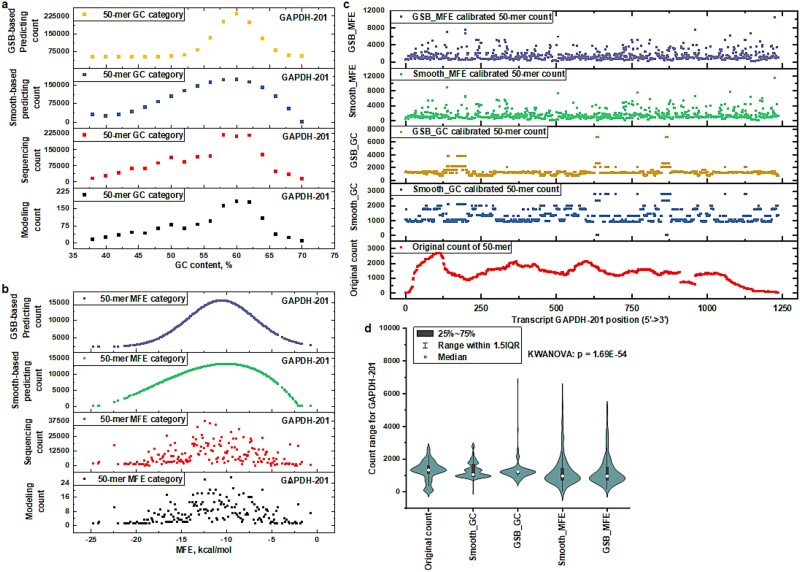
Comparison of sequencing count calibration between the GSB framework and smoothing method. (a) The stack plot presents a comparison across all categories of GC content for four different metrics: the modeling count, the original sequencing count, the count predicted via smoothing-based methods, and the count predicted through GSB-based methods, specifically focusing on the GAPDH-201 transcript (ENST00000229239). b) The stack plot showcases a comparative analysis across all MFE categories for the GAPDH-201 transcript. It includes four distinct metrics: the modeling count, the original sequencing count, the count predicted using smoothing-based techniques, and the count predicted through GSB-based methods. (c) The stack plot presents a detailed comparison of the original sequencing counts for each 50-mer across the GAPDH-201. It showcases predicted counts based on GC content using both smoothing techniques and the GSB method, alongside predictions made through MFE smoothing and the MFE-GSB approach. (d) The distribution of individual counts obtained through different methodologies. Assessment of variance in individual counts across different methodologies using the Kruskal-Wallis analysis of variance (KWANOVA) test.

To enhance the validation of the MFE-GSB framework designed for mitigating joint biases, an extensive comparative study was conducted. This study focused on various classifications grounded in GC content and MFE. The analysis utilized both smoothing-based techniques and the GSB framework, concentrating on key metrics: observed sequencing counts, counts predicted by smoothing methods, and counts estimated by the GSB framework. The evaluation employed GC content-based approaches, incorporating both GSB and smoothing methods, specifically aimed at addressing bias mitigation in the Glyceraldehyde-3-phosphate dehydrogenase (GAPDH)-201 transcript template (as illustrated in Fig. 4a). A Gaussian distribution function was applied to model counts categorized by GC content, allowing for the predetermination of essential parameters (as illustrated in the supporting information Fig. S7 available online at http://bib.oxfordjournals.org). A stack plot offered comprehensive comparisons among various data sets, including modeling counts categorized by GC content with an even 50-mer distribution, original sequencing counts categorized by GC content, counts calibrated through GC-based smoothing, and predicted counts utilizing GC-based GSB analysis. This visualization yielded deep insights into the different methodologies employed. In this framework, the counts of the GAPDH-201 transcript were segmented into 17 unique categories based on their GC content. For each category, count predictions were made using both smoothing-based and GSB-based techniques, facilitating the calibration of biased data. The study was further extended to organize counts according to MFE, culminating in 177 distinct categories. Initially, a Gaussian distribution function was utilized to model counts categorized based on MFE rather than GC content. This allowed for the advanced determination of critical parameters for an MFE-GSB framework (as detailed in the supporting information Fig. S8 available online at http://bib.oxfordjournals.org). Predictions of sequencing counts were made for each of these categories using both smoothing and GSB-based approaches (as showcased in Fig. 4b). The categorization based on MFE introduces a considerably wider range of structural variations than categorization solely by GC content, fostering a more detailed understanding of the variability in sequencing counts. This approach significantly enhances the characterization of *k*-mer count distribution, thereby improving bias mitigation accuracy in the analysis of sequencing data.

The counts predicted by both the GC and MFE-based smoothing methods or GSB framework are further averaged for each individual 50-mer within GAPDH-201. A stacked plot that includes the original sequencing count, counts adjusted by the GC-based method with smoothing, counts adjusted by the GC-based method with the GSB framework, counts adjusted by the MFE-based method with smoothing, and counts adjusted by the MFE-based method with the GSB framework is presented (as shown in Fig. 4c). The distribution of counts for individual 50-mers across different adjustment methods shows distinct variations (as illustrated in Fig. 4d). Notably, compared to the GC-based method, calibrating at the single 50-mer level using the MFE-based approach can realign the distribution of individual counts for more uniform patterns. Furthermore, transitioning from MFE-based smoothing to the MFE-GSB framework yields a reduction in SD, indicating more consistent data outcomes. Critically, the aggregation of counts within each MFE category in the MFE-GSB framework aligns perfectly with the Gaussian distribution pattern. This is a stark contrast to the outcomes of using the MFE- or GC-smoothing method alone, which fails to align count aggregates closely with the well-fitted Gaussian distribution (as illustrated in the supporting information Fig. S9 available online at http://bib.oxfordjournals.org).

### Comparative analysis of minimum free energy–based Gaussian Self-Benchmarking and other bias correction methods

**Fig. 5:**
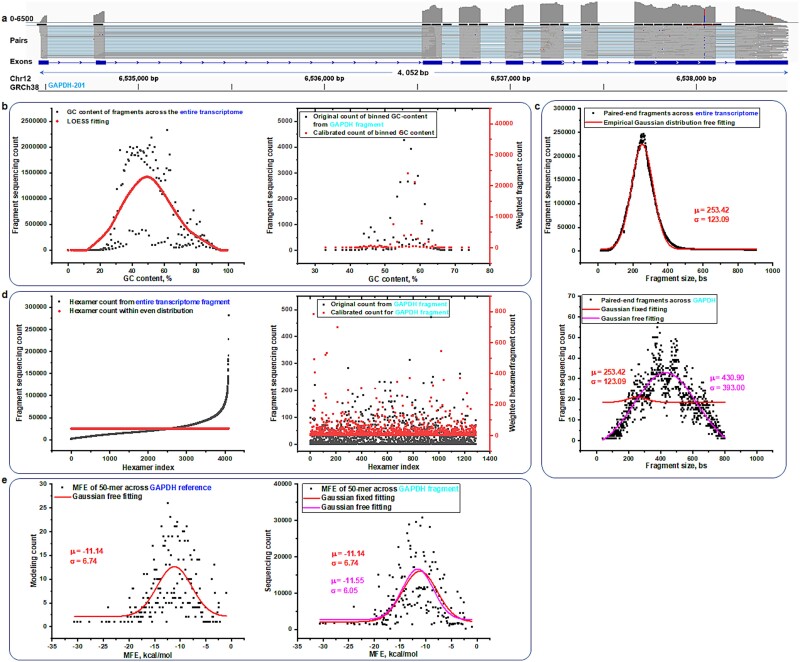
Validation and correction of different types of biases in RNA-seq. (a) Visualization of sequencing depth across nine exons of the GAPDH-201 transcript, highlighting variations in read coverage for paired-end fragments along the genomic reference. (b) GC bias correction methodology establishes a relationship between the 0.5-interval binned GC content of fragments and their sequencing counts across all transcripts using empirical data and then applies GC-content-dependent weighting factors to calibrate fragment counts for GAPDH-201. (c) Fragment size bias correction approach utilizes an empirical Gaussian distribution fitted to the size distribution of fragments from all transcripts in sequencing data to determine key parameters, which are then used to calibrate fragment counts for GAPDH. bs: bases. (d) Hexamer binding bias correction analyzes correlations between sequencing counts and each of the 4096 hexamers as the initial six bases of fragments from empirical data, applying calculated weighting factors to adjust GAPDH-201 fragment counts that initiate with each specific hexamer. (e) MFE-GSB framework demonstrates a theoretical model-based approach using predetermined Gaussian distribution parameters to calibrate sequencing counts for 50-mer fragments across various MFE categories, independent of empirical data.

To assess the effectiveness of the GSB model, it is crucial to perform a comparative analysis alongside other widely used bias correction methodologies. Notable transcript quantification tools like Salmon, Cufflinks, and Kallisto incorporate specific strategies to tackle various biases—GC bias [15], fragment bias [16], and hexamer priming [20], respectively. Therefore, our comparison will involve evaluating the GSB framework against these distinguished methods, each known for its unique approaches to bias mitigation. For a clear examination of how these biases might affect sequencing coverage unevenly across a reference genome, consider the example of the GAPDH-201 gene, which consists of nine exons. When sequencing depth is plotted while aligning paired-end fragments, one can easily observe variations in read coverage across different genomic regions (as shown in Fig. 5a). This plot unveils substantial discrepancies in the sequencing coverage throughout the gene’s sequence. This GAPDH dataset is set to undergo comparison using various bias mitigation models to ensure a comprehensive evaluation. Moreover, the analysis underlines significant base-calling errors, particularly around the eighth exon (as illustrated in supporting information Fig. S10 available online at http://bib.oxfordjournals.org). This error, evident at this specific site, notably affects the *k*-mer sequencing results and is highlighted by a marked reduction in the 50-mer sequencing count profile throughout the transcript (as depicted in Fig. 4c, bottom). This visualization and analysis help articulate how specific biases can influence genomic sequencing outputs and the importance of employing effective bias correction methods like the MFE-GSB model.

Addressing GC content biases is crucial for rectifying imbalances in fragment counts stemming from variations in the GC content among different transcript fragments. One effective method for this correction is LOESS (Locally Estimated Scatterplot Smoothing), a nonparametric regression technique that adeptly smooths the relationship between GC content and fragment count throughout the transcriptome [24]. Despite its benefits, directly applying LOESS to raw data poses challenges, as evidenced by the suboptimal fit quality when utilizing original GC content data from various lengths of paired-end fragments (refer to supporting information Fig. S11 available online at http://bib.oxfordjournals.org). To enhance the fit quality, we implemented a strategy of organizing the GC-indexed fragment count data into bins (as illustrated in Fig. 5b). This organization helps mitigate the complex variability in GC content across different fragment lengths, thus improving the accuracy of the LOESS fit. It also provides robust and global weighting factors for each GC content category (as depicted in supporting information Fig. S12 available online at http://bib.oxfordjournals.org). In cases like the GAPDH transcript, we adjust sequencing fragment counts by applying a weighting factor to neutralize GC bias. Although this adjustment corrects the counts, it introduces additional variability in the calibrated data across various GC categories, indicating a need for further refinement of the calibration process.

Fragment size bias is another challenge in RNA-seq, where certain fragment lengths may be preferentially amplified, sequenced, or aligned due to biases in the methodologies and enzyme preferences used. To tackle this, researchers typically examine the distribution of all mapped fragments across the transcriptome to identify an overarching pattern [16, 26]. In our analysis, we pinpointed an empirical Gaussian distribution of paired-end fragment sizes (as shown in Fig. 5c). This global distribution provides key parameters that define the overall behavior of fragment sizes across the transcriptome. Using this Gaussian model, we then adjust fragment counts for specific transcripts such as GAPDH. These adjustments are made to mitigate discrepancies stemming from the unequal representation of different fragment sizes. Furthermore, we use a cumulative distribution function (CDF) derived from a Gaussian model to establish key calibration parameters that account for the cumulative impact of fragment counts across different fragment sizes within the transcriptome. By setting these parameters, we calibrate the fragment counts of GAPDH to match the predefined CDF. This calibration process yields an amplitude from the fitting method, which represents the adjusted count (as illustrated in the supporting information Fig. S13 available online at http://bib.oxfordjournals.org). Despite these corrective measures, there are still notable differences between the fragment size distribution of GAPDH and that of the entire transcriptome. This suggests that the fixed-parameter alignment of GAPDH data aims to standardize the fragment size data across all transcripts, but it does not specifically address the issue of fragment size bias.

Hexamer priming bias also affects RNA-seq, wherein random hexamer primers exhibit preferential binding to certain RNA sequences during sample preparation, skewing the representation of RNA types in sequence data. To counteract this, the frequencies of each hexamer appearing at the start of reads or fragments across the transcriptome are analyzed [17]. Mitigation involves setting a theoretical even distribution as a baseline for all hexamers (as demonstrated in Fig. 5d). Each hexamer’s observed count is then standardized against this average to compute a weighting factor (as illustrated in the supporting information Fig. S14 available online at http://bib.oxfordjournals.org). These factors adjust the read counts starting with each hexamer by applying the respective weighting factor, thus correcting for hexamer-related biases in specific transcripts, including GAPDH. This method ensures that adjustments are made precisely for the detected bias. Nevertheless, it is crucial to recognize that these weighting factors are unique to each sample and can be influenced by various external variables.

In summary, the strategies to address biases like GC content, fragment size, hexamer binding, and other types of biases fundamentally depend on empirical sequencing data to develop their initial models [17, 21–24]. However, it is unlikely to find a dataset that is biased in just one specific way, as real-world data often exhibit a variety of interconnected biases. Consequently, when developing weighting factors or parameters intended to counteract one specific bias, they might inadvertently interact with other biases present within the data due to these inherent complexities. This complexity highlights the difficulty in achieving completely unbiased RNA-seq data. Unlike these methods, the MFE-GSB framework operates independently from empirical data, utilizing theoretical modeling data (as shown in Fig. 5e) instead. This approach allows for the predefined setting of key parameters using an MFE-based Gaussian distribution and its CDF. By fixing these parameters, the CDF can be applied to fit the MFE-categorized sequencing data to accurately quantify abundance levels, such as those of GAPDH (as illustrated in the supporting information Fig. S15 available online at http://bib.oxfordjournals.org). These theoretical data-driven key parameters accurately reflect the actual characteristics of the transcript without any external interferences, potentially leading to truly unbiased sequencing data for downstream analyses.

### Optimizing abundance calibration in transcript-specific samples using the minimum free energy–based Gaussian Self-Benchmarking approach

**Fig. 6:**
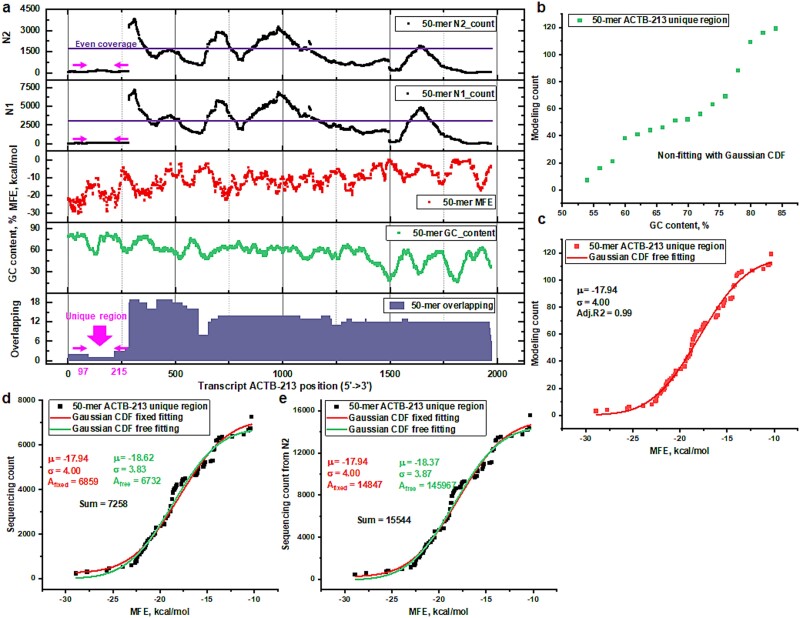
Enhancing the validation of MFE-GSB across diverse scenarios. (a) The sequencing of 50-mer counts along the ACTB-213 isoform (ENST00000642480) from two human sample replicates, including MFE, GC content, and the degree of overlap with other isoforms, is presented in a stacked plot. The unique region for ACTB-213 is highlighted. (b) The aggregate count of 50-mer sequences across unique region from ACTB-213 is organized based on their GC content. (c) The cumulative aggregates of 50-mer count from the unique region of ACTB-213 is organized through categorization based on their MFE. A CDF is used to model these MFE-classified cumulative counts, offering the key parameters. (d, e) The MFE-GSB framework, which uses a parameter-fixed CDF, is employed to calibrate cumulative 50-mer sequencing counts across two replicates. This process entails a comparative analysis of three different data sets: the original cumulative counts, the cumulative counts adjusted via a free-fitting method, and the cumulative counts calibrated using a fixed-parameter method.

The MFE-GSB framework offers a sophisticated approach for accurately calibrating biased counts, despite confronting the significant challenge posed by segments shared among multiple isoforms. The inherent complexity of these shared segments results in inconsistent overlap patterns, which do not support transcript-specific bias data calibration due to the absence of transcript-specific counts for analysis. In an effort to resolve the challenge of specifically identifying transcript isoforms, a few researchers have introduced a method where a whole transcript entity is represented by a unique segment [27–29]. Drawing inspiration from this innovative strategy, MFE-GSB prioritizes the analysis of isoforms through their unique regions, aiming to achieve precise transcript-specific count calibration. In this research, we implemented a GSB calibration focusing on the 50-mer sequences derived from the 5′-start position of the unique region in the human ACTB-213 transcript (as illustrated in Fig. 6a). The visualization incorporates components such as sequencing counts of 50-mers, the degree of isoform overlap, GC content, and the MFE, arranged in a stacked plot. Regions with an isoform overlapping degree of one denote unique areas specific to that particular transcript isoform. Both sets of our replicate samples showcased a consistent distribution pattern of the 50-mer counts along the entire transcript length, including its unique region (as detailed in supporting information Fig. S16 available online at http://bib.oxfordjournals.org). Further analysis through linear regression (as illustrated in supporting information Fig. S17 available online at http://bib.oxfordjournals.org) highlights a stable and consistent correlation between sequencing counts and the intrinsic structure of the RNA template. This finding points to the critical role that understanding the complex architecture of the RNA template plays in identifying the reasons behind differences in sequencing efficiency across distinct transcript templates.

Our study organizes *k*-mer modeling counts from this unique region and employs the GSB framework to calibrate each 50-mer’s count. To account for the overall sequencing abundance of *k*-mers from this region, we introduce a Gaussian CDF, developed directly from the Gaussian distribution based on MFE. This advancement allows the GSB framework, enhanced with the CDF, to adeptly adjust the cumulative *k*-mer counts. The evenly distributed modeling 50-mer counts from unique region does not lend itself to CDF fitting when the counts are organized by GC content (as demonstrated in Fig. 6b). This observation highlights a significant limitation in applying the GC-based GSB approach. In contrast, the MFE-categorized counts can be fitted very well with Gaussian CDF (as demonstrated in Fig. 6c). By predetermining essential parameters, the GSB framework efficiently employs the Gaussian CDF to model cumulative *k*-mer counts across MFE-diverse categories. This detailed procedure produces an amplitude profile, acting as a crucial indicator of unbiased abundance and offering a comprehensive portrayal of the unique region’s *k*-mer contributions (as shown in Fig. 6d and e). The calibration abundance obtained can be further refined by normalizing it to reads per kilobase of transcript per million mapped reads or transcripts per million, which are commonly used units for quantifying transcript abundance. Importantly, MFE-GSB identifies critical parameters independently from the sequencing data themselves, which ensures the elimination of inherent data biases and accurately reflects the *k*-mers’ true abundance contributions. Moreover, we perform a comparative analysis between a flexible fitting of cumulative *k*-mer counts and a fixed-parameter methodology. The comparison underscores significant distinctions in amplitude outcomes from these strategies and their *k*-mer count summations. These differences confirm our approach’s effectiveness in achieving an impartial evaluation of *k*-mer abundance, successfully navigating around potential biases for a thorough and precise analysis.

## Discussion

The MFE metric significantly refines our understanding of RNA’s two-dimensional (2D) structural stabilities, moving well beyond the rudimentary one-dimensional (1D) indicators, such as GC content. By incorporating Gaussian distribution models that are enhanced with insights derived from MFE analysis, this MFE-GSB framework reveals critical details. It shows how the sophisticated 2D configurations of RNA structures can influence sequencing efficiency, thereby introducing biases in the sequencing data. Importantly, the text skillfully points out the subtle differences among *k*-mers that, although they may have similar GC compositions, display significant dissimilarities in their 2D structural formations. These differences in 2D structures play a crucial role in affecting sequencing counts, highlighting the importance of recognizing these nuanced distinctions among *k*-mers. This level of detailed analysis is crucial for effectively identifying and addressing biases that arise from variations in sequencing efficiency. Such biases are particularly prevalent in scenarios where there are limited, unique minimal-shared *k*-mers specific to certain transcripts. The MFE-GSB framework could play a pivotal role in this context, offering a precise methodology for calibrating data. The MFE-GSB method’s ability to pinpoint these minor distinctions not only augments the accuracy of bias mitigation but also deepens our insight into the complex relationship between RNA structure, its sequencing efficiency, and its biological function.

Comparing MFE to GC content reveals a more nuanced understanding of *k*-mer structures. For example, when categorizing 65 536 unique spike-in RNA sequences, GC content allows for their classification into only nine distinct groups, whereas MFE analysis distinguishes up to 120 types. Taking specific sequences as case studies, the GAPDH-201, which includes 1236 50-mers, shows 17 different classifications based on GC content but expands to 177 when using MFE as the criterion. Similarly, the ACTB-213 sequence, which contains 1972 50-mers, is categorized into 35 groups by GC content, but MFE analysis enhances this distinction by identifying 258 unique types. These examples illustrate that MFE provides a significantly higher resolution of RNA structure compared to GC content, indicating its powerful ability to differentiate between RNA sequences more precisely. However, it’s important to recognize that this MFE-based method does not achieve the ultimate goal of single *k*-mer resolution. While the technique of averaging predicted counts in MFE-GSB comes close by providing detailed analysis at the level of individual 50-mers, calibration is still performed based on the MFE categories. Therefore, despite the increased detail offered by MFE analysis, the granularity it provides is limited to categorization into MFE groups, rather than identifying unique characteristics at the single 50-mer level.

Addressing biases in RNA-seq necessitates an in-depth exploration of their intrinsic sources. The complex, multidimensional structures of RNA molecules may be key to unraveling the mechanisms behind these biases [30, 31]. Although measures like GC content and MFE shed light on 1D and 2D aspects, they fall short of capturing the intricate functional architectures of RNA fragments. The distinct 3D structures of RNA should have a profound impact on its sequencing efficiency, yet accurately quantifying the 3D attributes of RNA fragments with diverse lengths and sequences remains challenging [32]. At present, a direct correlation between sequencing coverage and 3D structural features is elusive. To tackle this challenge, our approach involves identifying key parameters to characterize the 3D structural properties of RNA fragments. We plan to leverage machine learning/deep learning techniques to craft a model capable of predicting sequencing biases based on the 3D structural features of *k*-mers throughout the transcript. This forward-thinking strategy promises to shed light on how the functional structural characteristics of RNA intersect with sequencing coverage biases. Enhancing our understanding and handling of these biases in RNA-seq data could significantly improve both the accuracy and the interpretability of the results, marking a step forward in the field.

Based on the MFE-GSB framework, we have developed a new quantification platform called MFE-GaussF. This platform is designed to streamline the process of quantifying transcript abundance, taking raw sequencing data as input and producing transcript-associated abundance as output. MFE-GaussF is currently undergoing testing with disease-related RNA-seq data, with a particular focus on isoform-level quantification. While MFE-GSB broadly addresses bias-correcting model construction and mechanism exposition, MFE-GaussF is specifically tailored for practical applications, aiming to quantify transcript abundance accurately within an integrated system. We are currently refining the coding on this new platform. The figure (in supporting information Fig. S18 available online at http://bib.oxfordjournals.org) illustrates how the MFE-GaussF platform operates. By developing and focusing on MFE-GaussF, we aim to offer a more integrated and seamless approach to RNA-seq analysis, leveraging the strengths of the MFE-GSB framework to enhance RNA-seq utility and accuracy. This is part of our continued commitment to advancing the field of RNA sequencing analysis by addressing existing challenges and providing innovative solutions.

The validation of the MFE-GSB bias correction method is crucial for assessing its performance. The MFE-GSB approach is tailored to effectively characterize isoforms by focusing on regions that are minimally shared among them. It employs two main strategies: isolating unique regions specific to a single isoform and identifying minimal-shared regions across multiple overlapping isoforms. The first approach isolates a region unique to a single isoform, which acts as a definitive marker for that isoform. The second strategy identifies the smallest set of overlapping regions shared among various isoforms, thereby reducing representation ambiguity. An illustration of this approach is provided in Supporting Information Fig. S19 available online at http://bib.oxfordjournals.org, which examines the ACTB-215 isoform. According to the MFE-GSB-based MFE-GaussF method, this isoform registers a null sequencing count. In contrast, several other isoform quantification platforms detect this isoform with relatively high abundance values. This discrepancy highlights significant variations in isoform quantification methodologies. Traditional methods typically rely on aligning reads along the full transcript length. However, the MFE-GSB MFE-GaussF method adopts a different strategy, focusing instead on unique and minimally shared regions. To address these methodological differences, we conducted a comprehensive comparison of isoform abundance determination approaches. The results, detailed in Fig. 6 and Supporting Information Fig. S19 available online at http://bib.oxfordjournals.org, suggest that though the MFE-GSB MFE-GaussF method has its limitations and may occasionally fit unsuitably, it generally performs adequately. Furthermore, we are currently extending this approach in a transcriptome-wide evaluation project, where we have analyzed Huntington’s disease sequencing data using our MFE-GSB-based MFE-GaussF method. This analysis aims to identify differentially expressed isoforms, which are subsequently targeted in knockdown experiments to explore the underlying mechanisms of Huntington’s disease. This ongoing research demonstrates the potential of our method to contribute valuable insights into gene regulation and disease pathology.

**Key Points**

- Algorithm
- Self-benchmarking
- Bias

## Supplementary Material

BiB_supplementary_materials_revised_20241008_bbae532

## Data Availability

The raw and processed sequencing data have been deposited in the NCBI Sequence Read Archive (SRA) and can be publicly accessed via the following link: https://www.ncbi.nlm.nih.gov/sra. To locate these datasets, please use the project accession number PRJNA999048. Specifically, RNA sequencing (RNA-seq) data for samples labeled N1 and N2 are available under the accession numbers SRR25438891 and SRR25438890, respectively. For samples N8, which include 50-mer spike-ins across both the first and second replications, the relevant accession number is SRR25438885. Furthermore, the accession numbers for the HEK293T total RNA-based RNA-seq data, for both the first and second replications, are SRR25438884 and SRR25438883, respectively. Should there be a need for additional information or data, we encourage requests to be directed to the corresponding authors.

## Code availability

The code used for data analysis is available at https://github.com/QiangSu/MFE-GSB. The code is also available in methods.
