# Supplementary material for: Enhancing RNA-seq analysis by addressing all co-existing biases using a self-benchmarking approach with 2D structural insights
Source: Brief Bioinform. 2024 Oct 19;25(6):bbae532. doi: 10.1093/bib/bbae532 (PMC11491153; doi:10.1093/bib/bbae532)
Supplement: BiB_supplementary_materials_revised_20241008_bbae532 [file bib_supplementary_materials_revised_20241008_bbae532.docx]

Supplementary Materials for

Enhancing RNA-seq Analysis by Addressing All Co-Existing Biases Using a Self-Benchmarking Approach with 2D Structural Insights

Qiang Su^1,4,‡,*^, Yi Long^2,‡^, Deming Gou^3^, and Junmin Quan^4,*^, Qizhou Lian^1,5,6,*^

^1^Faculty of Synthetic Biology, Shenzhen University of Advanced Technology, Shenzhen; Key Laboratory of Quantitative Synthetic Biology, Shenzhen Institute of Synthetic Biology, Shenzhen Institutes of Advanced Technology, Chinese Academy of Sciences, Shenzhen, China

^2^Institute of Chemical Biology, Shenzhen Bay Laboratory, Shenzhen, China

^3^Shenzhen Key Laboratory of Microbial Genetic Engineering, Vascular Disease Research Center, College of Life Sciences and Oceanography, Shenzhen University, Shenzhen, China.

^4^State Key Laboratory of Chemical Oncogenomics, School of Chemical Biology and Biotechnology, Peking University Shenzhen Graduate School, Shenzhen, China

^5^Cord Blood Bank, Guangzhou Institute of Eugenics and Perinatology, Guangzhou Women and Children’s Medical Center, Guangzhou Medical University, Guangzhou, China

^6^State Key Laboratory of Pharmaceutical Biotechnology, and Department of Medicine, the University of Hong Kong, Hong Kong SAR, China.

**This file includes:**

Theoretical consideration

METHODS

Figs. S1 to S19

**Theoretical consideration**

The theoretical foundation and empirical evidence supporting the MFE-GSB algorithm are based on employing the binomial distribution model of GC content as a statistical framework. This model excels in calculating the likelihood of observing a certain quantity of successes within a given number of independent events. Its effectiveness is particularly highlighted when assessing the GC content in a specified section, or bin size, (k) of an RNA sequence. In this scenario, each nucleotide position across the RNA strand is treated as an individual trial that can result in one of two outcomes (as depicted in Fig. 1a): a success, indicated by the occurrence of a degenerate base (S) (either G or C), or a failure, marked by the appearance of a base (W) (A or U). In this model, the probability of encountering a degenerate base (S) at any position is represented by (p), and the likelihood of finding a base (W) is expressed as (q = 1 - p). For analytical purposes, the RNA fragments are viewed as sequences of n independent Bernoulli trials, each with a success probability of p = 1/2 for landing a GC base. Let X represent the random variable that denotes the count of successful GC base assignments in these n trials (where n=k). To derive the probability mass function (PMF) of X, one must calculate the likelihood of achieving exactly X successes. This calculation involves raising the success probability, p, to the power of X (p^X), and raising the failure probability, (1-p), to the power of (n-X) ((1-p)^(n-X)). The binomial coefficient, which calculates the number of different ways X successful GC assignments can occur among n trials, is determined as follows:

$C_{n}^{X}=\frac{n!}{X!*\left( n-X \right)!}$ (Eq.1)

Thus, the PMF of X integrates these elements:

$P_{X}=\frac{n!}{X!*\left( n-X \right)!}*p^{X}*q^{\left( n-X \right)}=\frac{n!}{X!*\left( n-X \right)!}*{(\frac{1}{2})}^{n}$ (Eq.2)

This formula seamlessly integrates the binomial distribution model to evaluate GC content in RNA fragments, offering a robust method for determining the likelihood of encountering different GC content levels within this specific framework.

Utilizing the binomial distribution offers a precise and effective means to study the distribution of GC-content across RNA fragments of defined length, through the pseudo-random arrangement of G and C bases. However, it's worth noting that the binomial distribution, with its unchanging parameters, is inherently suited for modeling results across a constant number of trials (n), mirroring a uniform RNA fragment length. Nevertheless, its utility is limited in scenarios dealing with mixed RNA populations or under conditions characterized by variability. To address this constraint, a Gaussian function has been adapted from the fundamentals of the binomial distribution. Specifically, when the size of the binomial distribution's parameters-n (total trials), np (average successes), and nq (average failures)-grows substantially, the approximation of the binomial distribution by the Gaussian distribution becomes remarkably accurate, following the de Moivre-Laplace theorem.^26, 27^ This adaptation expands the analytical versatility, allowing for a broader investigation into the distribution of GC-content. This relation is articulated as follows:

$f_{\left( x \right)}=\frac{n!}{X!*\left( n-X \right)!}*p^{X}*\left( 1-p \right)^{\left( n-X \right)}\sim\frac{1}{\sqrt{2\pi npq}}e^{-\frac{{(x-np)}^{2}}{2npq}}=\frac{1}{\sqrt{2\pi}\sigma}e^{-\frac{{(x-\mu)}^{2}}{2\sigma^{2}}}$ (Eq.3)

The Gaussian distribution function significantly enhances the flexibility and utility of data analysis, offering a sophisticated approach to understanding a wide range of experimental phenomena that surpass the limited scope of binomial distributions. In the realm of evaluating GC distribution patterns across various RNA fragments, this adaptability proves especially valuable, facilitating a more nuanced and adaptable analytical perspective. Building upon this foundation, the Gaussian distribution function has been expanded from its traditional application of denoting probabilities to also encompass the concept of frequency or occurrence rates. This pivotal shift involves the transformation of the Probability Mass Function (PMF) into an Occurrence Mass Function (OMF). To accomplish this, the PMF is multiplied by the total number of observations, 'N', within a specific transcriptomic dataset. In this context, 'N' represents the comprehensive tally of categorized GC k-mers within the dataset. This revised formulation is denoted as:

$f_{occu\_GC\left( x \right)}=A*\frac{1}{\sqrt{2\pi}\sigma\_GC}e^{-\frac{{(x-\mu\_GC)}^{2}}{2{\sigma\_GC}^{2}}}$ (Eq.4)

Deriving a MFE-GSB model from a GC content-based Gaussian framework necessitates a sophisticated level of abstraction that acknowledges the intricate two-dimensional architecture of RNA molecules. The MFE metric offers a crucial quantitative assessment of the stability inherent in RNA secondary structures, which significantly impacts the molecular functions of RNA. To develop an MFE-GSB model, we embark on a series of theoretical stages, building upon the core principles of the GC-based Gaussian model. The first step entails recognizing the fundamental relationship between GC content and the MFE of RNA structures. GC pairs, which form through three hydrogen bonds, contribute more robustly to the stabilization of RNA secondary structures than AU pairs, which form through only two hydrogen bonds.^28^ This significant difference results in a general trend where an increase in GC content correlates with a decrease in MFE, indicating enhanced stability of the RNA structure. Given the complex nature of RNA secondary structure formation, this correlation can be succinctly represented through a sequence-specific linear modeling approach. In this framework, each RNA sequence, represented by subscript "i," is examined to understand how variations in its GC content (denoted as GCi) influence the corresponding MFE (represented as MFEi). The relationship between GC content and MFE for each sequence can thus be modeled linearly as follows:

MFEi = β0i + β1i * GCi (Eq.5)

Here, this model suggests that the MFE of an RNA sequence is a linear function of its GC content. Here, the coefficient β0i is the expected MFE value when the GC content is zero, indicating the baseline stability of the RNA structure. The coefficient β1i, which is anticipated to be negative, reflects the change in MFE for each unit increase in GC content. This negative correlation implies that a higher GC content results in a more stable RNA structure, as evidenced by a lower MFE value. Through this analysis, we propose transitioning from a GC-based model to an MFE-based model. ). The distribution of MFE values for a specific GC content can then be modeled using a Gaussian function:

$f_{occu\_MFE\left( x \right)}=A*\frac{1}{\sqrt{2\pi}\sigma\_MFE}e^{-\frac{{(x-\mu\_MFE)}^{2}}{2{\sigma\_MFE}^{2}}}$ (Eq.6)

The MFEs of sequences with similar GC content distribute around a mean MFE value (µ_MFE) with a certain standard deviation (σ_MFE).

**METHODS**

**Data analysis**:

1. Data processing. The software pipeline for processing raw paired-end data includes several essential tools for quality control, trimming, alignment, and analysis. Initially, FastQC (v0.11.8) by Babraham Bioinformatics assesses data quality. Subsequently, adapter sequences and low-quality bases are trimmed using Cutadapt (v2.10) and Trimmomatic (v0.39). The alignment of reads to the human reference genome GRCh38.p14 (Ensembl version) is conducted using HISAT2 (v2.2.1) and STAR (2.7.3a), ensuring accurate mapping against the genomic backdrop. Post-alignment operations are handled by Samtools (v1.7) and IGV (Integrative Genomics Viewer), facilitating the management of aligned reads for further analysis. The RSeQC package evaluates the RNA-seq data quality post-alignment. Furthermore, the pipeline aligns k-mer segments to transcript references as annotated in Ensembl, enhancing the depth of genomic analysis. Together, these tools streamline the data processing workflow, ensuring thorough preparation, alignment, and comprehensive genomic analysis.

2. Cutadapt for adapter removing. Cutadapt is a specialized tool tailored for the precise removal of adapter sequences. These sequences, short DNA fragments, are affixed to the ends of DNA sequences during the library preparation phase. Since various libraries utilize distinct adapters, it is crucial to remove them accurately to maintain sequencing data integrity. Cutadapt stands out by adeptly identifying and excising these adapter sequences, thereby vastly improving the sequencing results' quality and reliability.

2.1 N8 library:

for i in $(cat $path_raw_read/samplelist.txt)

do

cutadapt -j 5 -a CTGTCTCTTATACACATCTCCGAGCCCACGAGAC -A CTGTCTCTTATACACATCTGACGCTGCCGACGA -o ${path_raw_cutadapt}/${i}_cutadapt_R1.fq -p ${path_raw_cutadapt}/${i}_cutadapt_R2.fq $path_raw_read/${i}_raw_1.fq.gz $path_raw_read/${i}_raw_2.fq.gz

done

echo cut adapter done

2.2 293T library

for i in $(cat $path_raw_read/samplelist.txt)

do

cutadapt -j 5 -a AGATCGGAAGAGCACACGTCTGAACTCCAGTCAC -A AGATCGGAAGAGCGTCGTGTAGGGAAAGAGTGT -o ${path_raw_cutadapt}/${i}_cutadapt_R1.fq -p ${path_raw_cutadapt}/${i}_cutadapt_R2.fq $path_raw_read/${i}_raw_1.fq.gz $path_raw_read/${i}_raw_2.fq.gz

done

echo cut adapter done

2.3 N1-N2 human tissue library

for i in $(cat $path_raw_read/samplelist.txt)

do

cutadapt -j 5 -a AAGTCGGAGGCCAAGCGGTCTTAGGAAGAC -A AAGTCGGATCGTAGCCATGTCGTTC -o ${path_raw_cutadapt}/${i}_cutadapt_R1.fq -p ${path_raw_cutadapt}/${i}_cutadapt_R2.fq $path_raw_read/${i}_raw_1.fq.gz $path_raw_read/${i}_raw_2.fq.gz

done

echo cut adapter done

3. Trimmomatic for further cleaning data. Trimmomatic stands out as an exceptionally efficient tool dedicated to enhancing data quality. It primarily excels in three critical areas: eradicating adapter sequences, trimming bases of subpar quality, and purging the dataset of reads deemed low in quality. Through its meticulous refinement of reads, Trimmomatic significantly contributes to ensuring precise alignment and bolstering the reliability of subsequent analyses.

for i in $(cat $path_raw_read/samplelist.txt);

do

trimmomatic PE -threads 5 $path_raw_cutadapt/${i}_cutadapt_R1.fastq $path_raw_cutadapt/${i}_cutadapt_R2.fastq -baseout $path_raw_trimmomatic/${i}_cutadapt_trim.fastq LEADING:30 TRAILING:30 SLIDINGWINDOW:4:15 AVGQUAL:20 MINLEN:20

done

4.1 Alignment of cleaned reads to a reference sequence with HISAT2.

for i in $(cat $path_raw_read/samplelist.txt)

do

name=$(echo ${i%R1_001.fastq})

hisat2 -p 60 --dta -x /home/Ref/Hg19/hisat/hg19_tran -1 $path_raw_trimmomatic/${i}_cutadapt_trim_1P.fastq -2 $path_raw_trimmomatic/${i}_cutadapt_trim_2P.fastq -S ${sam_dir}/${i}_cutadapt_trim.sam

done

echo hisat2 done

4.2 Alignment of cleaned reads to a reference with STAR

4.2.1 STAR indexing

STAR --runMode genomeGenerate --runThreadN 5 --genomeFastaFiles ./Homo_sapiens.GRCh38.dna.primary_assembly.fa --genomeDir ./STAR_index/ --sjdbGTFfile ./Homo_sapiens.GRCh38.111.gtf --sjdbOverhang 149

4.2.2 STAR mapping

for i in $(cat $path_raw_read/samplelist.txt)

do

STAR --runThreadN 10 \

--genomeDir /home/boot/qiangsu/ref/STAR_index/ \

--readFilesIn /home/boot/qiangsu/drug_pool/TCM_process_data/trimmed_data/${i}_trim_1P.fastq /home/boot/qiangsu/drug_pool/TCM_process_data/trimmed_data/${i}_trim_2P.fastq \

--sjdbOverhang 149 \

--outFileNamePrefix /home/boot/qiangsu/drug_pool/TCM_process_data/sam_data/${i}- \

--outSAMtype BAM SortedByCoordinate \

--twopassMode Basic \

--quantMode TranscriptomeSAM GeneCounts \

--chimOutType Junctions SeparateSAMold \

--chimSegmentMin 10 \

done

5. Converting SAM files to BAM format using Samtools

for i in $(cat $path_raw_read/samplelist.txt)

do

samtools sort -@ 10 -o ${bam_dir}/${i}_cutadapt_trim.bam ${sam_dir}/${i}_cutadapt_trim.sam

done

echo samtools done

6. samtools index *.bam

for i in $(cat $path_raw_read/samplelist.txt)

do

samtools index -@ 10 ${bam_dir}/${i}_cutadapt_trim.bam

done

7. samtools extracting transcript-specific sequencing coverage

samtools view -h ${i}_cutadapt_trim.bam 11:77812752-77835555 | samtools view -Sb - > transcript-specified_chr11:77812752-77835555.bam

8. Outputting the length of paired-end determined fragments from BAM files.

samtools view 99272-N_raw_cutadapt_trim-chr12:13349660-13367806_EMP1-211.bam | awk '{print $8}' > 99272-N_raw_cutadapt_trim-chr12:13349660-13367806_EMP1-211.bam_fragment_end.txt

9. k-mer counting in kmer_counting_loop.py (<https://github.com/QiangSu/MFE-GSB>)

import argparse

import time

import multiprocessing

import os

import gzip

from collections import OrderedDict

import glob

from itertools import islice

def count_kmers(sequence, k, filter_set=None):

kmers = [sequence[i:i+k] for i in range(len(sequence) - k + 1)]

return kmers if filter_set is None else [kmer for kmer in kmers if kmer in filter_set]

def fastq_sequence_lines(file_path):

with gzip.open(file_path, 'rt') as fastq_file:

while True:

identifier_line = fastq_file.readline() # Skip the identifier line

if not identifier_line:

break # EOF

sequence_line = fastq_file.readline().strip() # Read and strip the sequence line

plus_line = fastq_file.readline() # Skip the '+' line

quality_line = fastq_file.readline() # Skip the quality line

yield sequence_line

def process_chunk(sequences, k, filter_set):

chunk_kmers = []

for sequence in sequences:

chunk_kmers.extend(count_kmers(sequence, k, filter_set))

return chunk_kmers

def main():

parser = argparse.ArgumentParser(description='Count k-mer frequencies in a FASTQ file.')

parser.add_argument('--k', type=int, help='Size of the k-mer.', required=True)

parser.add_argument('--chunk_size', type=int, help='Number of records processed per chunk.', required=True)

parser.add_argument('--fastq', type=str, help='Path to the FASTQ file.', required=True)

parser.add_argument('--kmer_dir', type=str, help='Directory containing input CSV files with k-mer sequences.', required=True)

parser.add_argument('--output', type=str, help='Output directory for storing CSV files.', required=True)

parser.add_argument('--threads', type=int, help='Number of threads to use for processing.', default=multiprocessing.cpu_count())

args = parser.parse_args()

k = args.k

chunk_size = args.chunk_size

fastq_file_path = args.fastq

kmer_dir = args.kmer_dir

output_directory = args.output

num_cores = args.threads

if not os.path.exists(output_directory):

os.makedirs(output_directory)

# Find all CSV files in the directory

kmer_files = glob.glob(os.path.join(kmer_dir, "*_kmers.csv"))

for kmer_csv_file_path in kmer_files:

kmers_from_csv = OrderedDict()

with open(kmer_csv_file_path, 'r') as csvfile:

for line in csvfile:

kmer = line.split(',')[0].strip()

if not kmer.lower().startswith("kmer") and kmer:

kmers_from_csv[kmer] = 0

csv_base_name = os.path.splitext(os.path.basename(kmer_csv_file_path))[0]

output_file_path = os.path.join(output_directory, f"{csv_base_name}_counts.csv")

start_time = time.time()

with multiprocessing.Pool(processes=num_cores) as pool:

chunk_results = []

for chunk_index in range(0, chunk_size*num_cores, chunk_size): # iterate over each chunk

sequences = list(islice(fastq_sequence_lines(fastq_file_path), chunk_index, chunk_index + chunk_size))

if not sequences:

break

chunk_result = pool.apply_async(process_chunk, args=(sequences, k, set(kmers_from_csv.keys())))

chunk_results.append(chunk_result)

for chunk_result in chunk_results:

chunk_kmers = chunk_result.get()

for kmer in chunk_kmers:

kmers_from_csv[kmer] += 1

with open(output_file_path, "w") as output_file:

output_file.write("K-mer,Count\n")

for kmer, count in kmers_from_csv.items():

output_file.write(f"{kmer},{count}\n")

end_time = time.time()

execution_time = end_time - start_time

print(f"k-mer count data for {csv_base_name} saved to {output_file_path}")

print(f"Execution Time: {execution_time:.2f} seconds")

if __name__ == "__main__":

main()

**Collection of tissue samples:** Colorectal samples were ethically obtained from Sun Yat-sen University Cancer Center and Shenzhen University General Hospital, with each donor granting informed consent prior to tissue collection. This consent facilitated the retrieval of biopsies and permitted comprehensive molecular profiling of their transcriptomes.

**Cell culture:** HEK293T cells were cultured in DMEM high glucose medium (HyClone, cat. no. SH30022.01), supplemented with 10% Fetal Bovine Serum (FBS, Thermo Fisher, cat. no. 10100147). The culture conditions were optimized, maintaining the cells in an incubator at 37°C with 5% CO_2 and saturated humidity to ensure optimal growth and viability.

**Library preparation:** The preparation of RNA-seq libraries for spike-ins, HEK293T cells, and colorectal samples adheres to the structured protocol provided by the VAHTS Universal V8 RNA-seq Library Prep Kit. This protocol encompasses essential steps including RNA fragmentation, cDNA synthesis using hexamer priming, end repair, the addition of an adenine to the 3' ends of DNA fragments (also known as dA-tailing), adaptor ligation, PCR amplification of the library, and its subsequent sequencing. For the spike-in samples, the protocol introduces a modification by employing a tagmentation step. This step serves as an efficient alternative to the traditional end repair, dA-tailing, and adaptor ligation steps, thereby streamlining the preparation process.

**RNA isolation:** Total RNA was extracted from cells using the RNAiso Plus kit (TaKaRa Biotechnology, catalog no. 9109), with strict adherence to the manufacturer's instructions. The extracted RNA was then dissolved in RNase-free water, a standard procedure followed for all RNA-related processes. To assess the quality of the RNA, a 2100 Bioanalyzer RNA picochip was employed. Subsequently, the RNA was aliquoted into 5 μg portions and stored at -80°C for future experimental use.

**rRNA depletion:** To accurately profile non-rRNA molecules in RNA samples, we employed the Ribo-off rRNA Depletion Kit (Human/Mouse/Rat) (kit no. N406-01, Vazyme), renowned for its efficiency in eliminating ribosomal RNA (rRNA) from the total RNA population. The rRNA reduction process is carefully designed, including several essential steps. Initially, the total RNA sample is mixed with specifically designed rRNA removal probes, which selectively target and bind to rRNA molecules. This mixture is subsequently incubated, allowing effective hybridization of the rRNA removal probes with the rRNA. Following incubation, a removal solution is introduced, facilitating the disassembly of the rRNA-probe complexes. The remaining RNA, now enriched with non-rRNA molecules, undergoes purification to isolate the desired RNA subset suitable for subsequent analyses. Utilizing the Ribo-off rRNA Depletion Kit plays a crucial role in reducing the rRNA content, thereby enhancing the comprehensive examination of non-rRNA molecules. This methodological improvement significantly elevates the sensitivity of subsequent RNA-seq techniques. Integrating an rRNA depletion step is critical for generating high-quality data, substantially improving the accuracy and reliability of our molecular research.

**Spike-in RNA and RNA Circularization:** To prepare the circular RNA spike-ins, a synthesized single-stranded RNA oligonucleotide was used. This oligonucleotide was designed with a 5' phosphate and a 3' hydroxyl end, and the sequence for the spike-ins was provided as follows: 5'-phosphate-AA AAAAAAGGTAACTGCGNTTANCACNAGCNCCANGAGNAACNACANGAATTCTTTATAAAAAAA-OH-3'. For the spike-in RNA preparation, 5 μL of this oligonucleotide, at a 10 μM concentration, was added to a reaction mixture. The mixture included 1 mM ATP and an Rnase inhibitor at 2 units/μL, along with 1 μL of T4 RNA ligase 1 (ssRNA Ligase) from New England Biolabs (Catalog No. M0204S), and PEG8000 to a final concentration of 50%, diluted to 15%. The ligation reaction proceeded by incubating the mixture at 25°C for 1-2 hours. Subsequently, the reaction was terminated by heating at 95°C for 2 minutes.

**Reverse transcription:** RNA samples, from which ribosomal RNA (rRNA) was removed, underwent reverse transcription utilizing random hexamers in conjunction with SuperScript™ IV reverse transcriptase (Invitrogen, Cat. No. 18090200) following the manufacturer's protocol. The process was carried out in a reaction volume of 20 μL, incorporating 100 ng of RNA template, 2.5 μM random hexamers, a dNTP Mix (with each dNTP at a concentration of 10 mM), and 200 units (equivalent to 1 μL) of SuperScript™ IV reverse transcriptase. The procedure began with an incubation period at 25°C for 10 minutes to facilitate primer binding. This was succeeded by the reverse transcription phase at 42°C for 50 minutes. The reaction was finally terminated by heating the mixture to 70°C for 15 minutes, a step which served to inactivate the reverse transcriptase enzyme.

**Tagmentation:** To carry out tagmentation, a combined mixture was prepared by adding 50 ng of DNA to a 30 μL reaction mixture, which consisted of 1× Insertion Buffer and 2 µL of Tn5-50 adaptor index (at a concentration of 10 µM). This mixture underwent an initial incubation at 55°C for 5 minutes. Subsequently, an additional 30 µL of 2× Tn5 Digestion Mix, procured from the TransNGS® Tn5 DNA Library Prep Kit for Illumina® (catalog no. KP101), was incorporated into the mixture. This augmented mixture was then subjected to a further incubation at 55°C for another 5 minutes. Through these steps, a tagmented DNA library was efficiently produced. The resulting tagmented library structure is as follows:

5'-AATGATACGGCGACCACCGAGATCTACAC-i5-TCGTCGGCAGCGTCAGATGTGTATAAGAGACAG-NNNNNN-CTGTCTCTTATACACATCTCCGAGCCCACGAGAC-i7-ATCTCGTATGCCGTCTTCTGCTTG-3'.

**PCR amplication:** The library amplification process utilized PCR, employing the 2× HIFI KAPA master mix. The specific mixture entailed combining 25 μL of the 2× HIFI KAPA master mix, 10 μL of cDNA, and 13 μL of H2O. To this, 1 μL of a primer was added—this could be either the universal forward or reverse primer, each at a concentration of 10 μM. The sequences of the primers, which incorporate phosphorothioate bonds for enhanced stability, are as follows:

Universal forward primer: 5'-AATGATACGGCGACCACCGAGATCTACACCTCTCTATACACTCTT-3'

Universal reverse primer: 5'-CAAGCAGAAGACGGCATACGAGATGTGACTGGAGTT-3'

The PCR amplification was performed in a thermal cycler following a specific sequence: an initial denaturation at 95°C for 5 minutes to separate the DNA strands. This initial step was succeeded by 10-15 cycles, consisting of two stages: a denaturation phase at 95°C lasting 15 seconds to further unwind the DNA, and an annealing/extension phase at 60°C for 30 seconds, allowing primers to bind and extend.

After the completion of the PCR amplification, the resulting library underwent a purification process using Ampure XP DNA Beads at a 1.8× concentration ratio. The purified library was then diluted in 20 μL of H2O. To assess the concentration and quality of the purified library, a 1 μL sample was analyzed using a Qubit 4 Fluorometer, in conjunction with the dsDNA HS (High Sensitivity) Assay Kit from Invitrogen.

**Sequencing:** The purified PCR libraries were subsequently sequenced using either the Illumina NovaSeq 6000 platform (PE150) or the MGISEQ-2000 platform (PE150). The choice between these platforms was determined by the specific data output yield requirements.

**Statistics:** To assess the effectiveness of linear and Gaussian functional fits, we employed Pearson correlation coefficients and adjusted R-squared values as primary metrics. These measures are key in identifying the strength and significance of the relationships depicted by the fits. For a more thorough evaluation, we further integrated parametric statistical methods, specifically the T-test, to enhance our analysis of the data. Additionally, we analyzed variance in individual counts across different methodologies using the Kruskal-Wallis ANOVA (KWANOVA) test to ensure a comprehensive understanding.

Fig. S1.


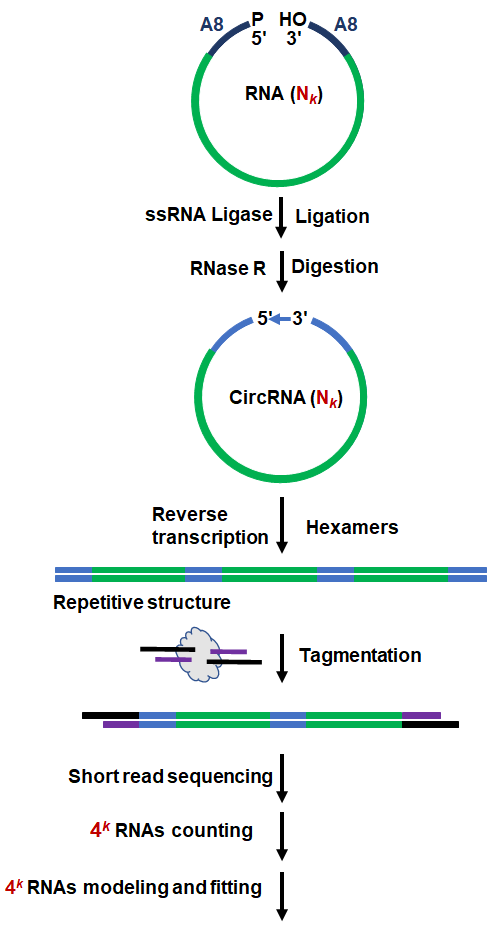


**Supplemental Fig. S1**. The stepwise process of RNA structure-associated RNA-seq. The N random nucleotides incorporated spike-in RNA string with two common A_8_ arms are ligated by single-strand RNA (ssRNA) ligase. Circular RNA templates facilitate rolling circular reverse transcription initiated by random hexamer priming. This is followed by the introduction of sequencing adaptors via tagmentation and amplification by PCR. Subsequently, the library is sequenced, and the data are analyzed.

Fig. S2.


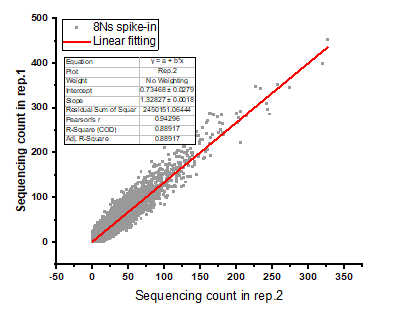


**Supplemental Fig. S2**. A linear regression analysis was conducted on data from biological replicates. This analysis entailed plotting the sequencing read counts for each of the 65,536 unique spike-in RNA templates, which were measured in duplicates. For this dataset, both Pearson's correlation coefficient and Spearman's rank correlation coefficient were computed to assess the relationship between the datasets.

Fig. S3.


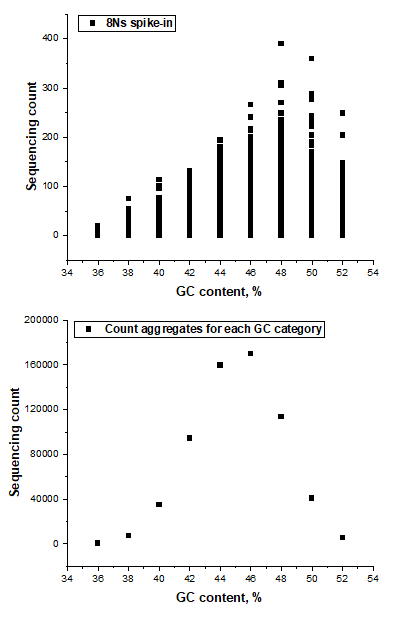


**Supplemental Fig. S3**. Aggregate the sequencing counts of individual spike-in RNA sequences, denoted as 4^8, by categorizing them according to their GC content, as shown in the upper panel. This approach reveals the distribution and collective tally of counts within various GC content categories, as detailed in the lower panel. It emphasizes the method of grouping sequences that share the same GC-content values, thereby illustrating the relationship between GC content and count distribution.

Fig. S4.


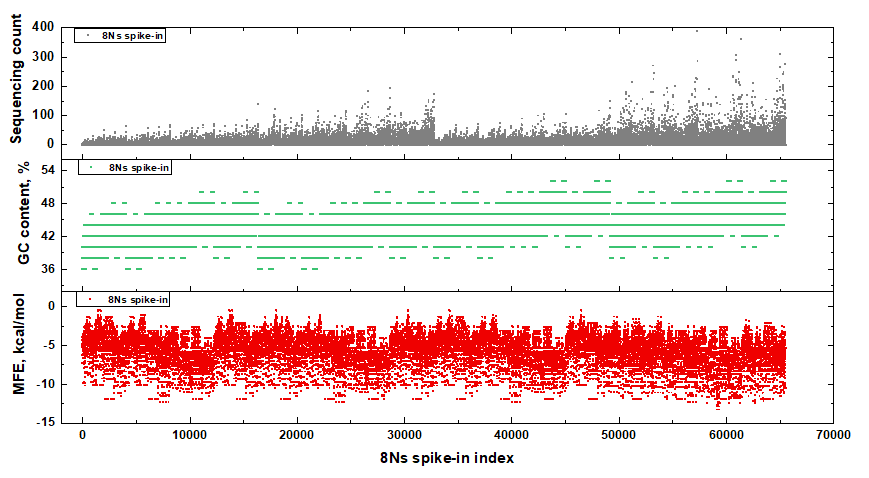


**Supplemental Fig. S4**. The examination of sequencing counts, GC content, and MFE values for all spike-in RNA template.

Fig. S5.


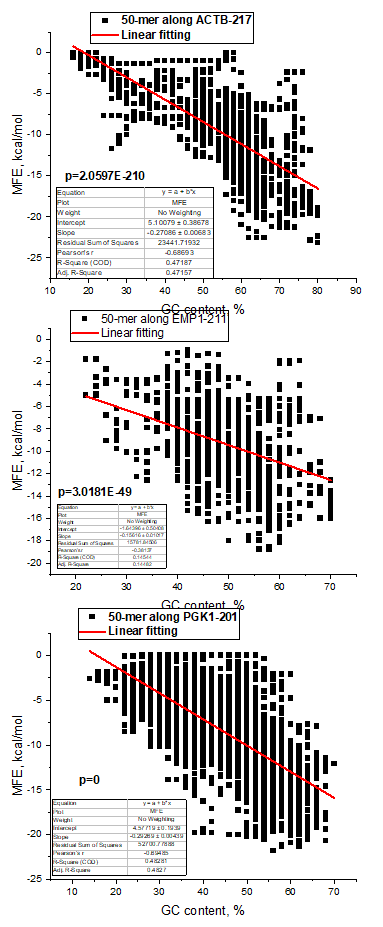


**Supplemental Fig. S5**. The linear regression analysis of 50-mer sequences from the ACTB, EMP1, and PKG1 genes uncovers an inverse linear relation, showing that a lower variability in GC content correlates with a higher variability in the MFE values.

Fig. S6.


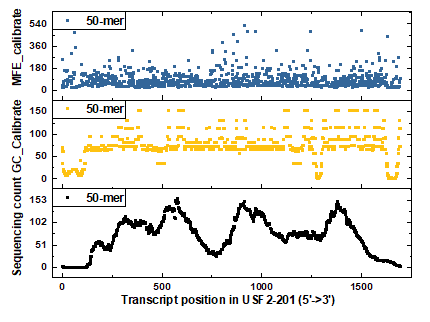


**Supplemental Fig. S6**. For each individual 50-mer along USF2-201 transcript, a precise level of correction is performed by comparing actual sequencing counts to those defined by the MFE-GSB and GC-based GSB benchmarks.

Fig. S7.


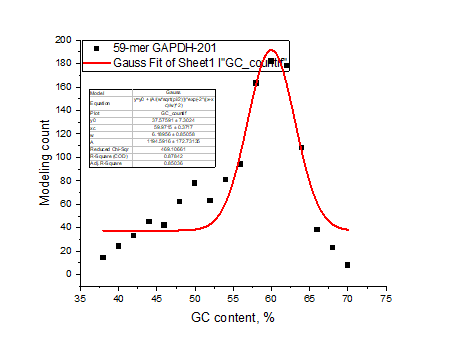


**Supplemental Fig. S7**. The modeling count aggregates for GAPDH-201, categorized by GC content, are analyzed using a Gaussian distribution function.

Fig. S8.


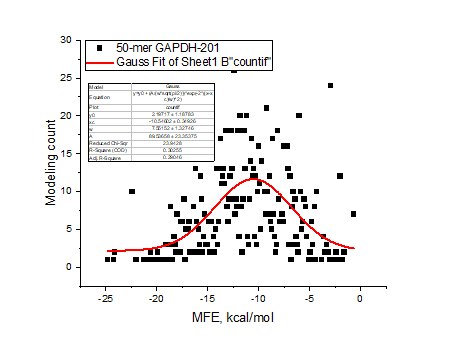


**Supplemental Fig. S8**. The modeling count aggregates for GAPDH-201, categorized by MFE, are analyzed using a Gaussian distribution function.

Fig. S9.


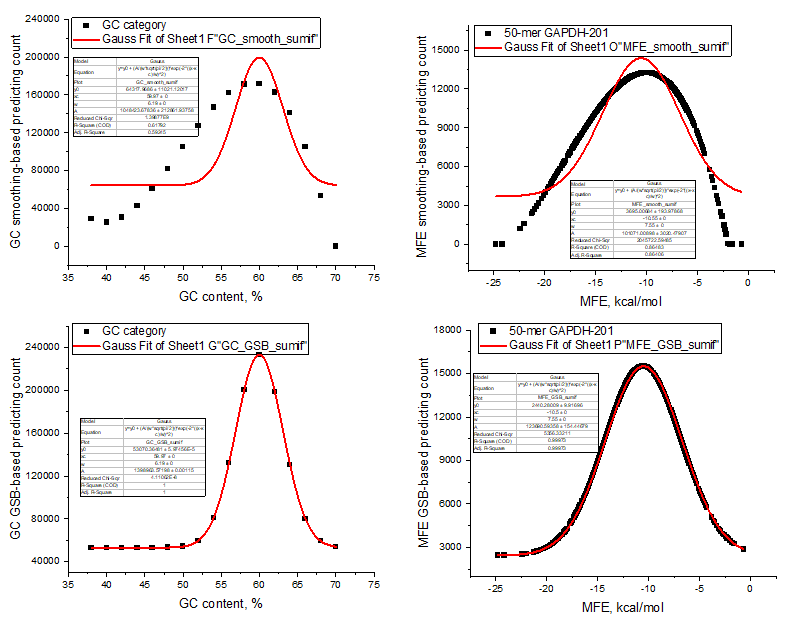


**Supplemental Fig. S9**. The Gaussian distribution function is utilized to fit the smoothing counts based on GC and MFE, as well as the predicting counts derived from GSB, across all categories of GC or MFE from GAPDH-201 transcript template.

Fig. S10.


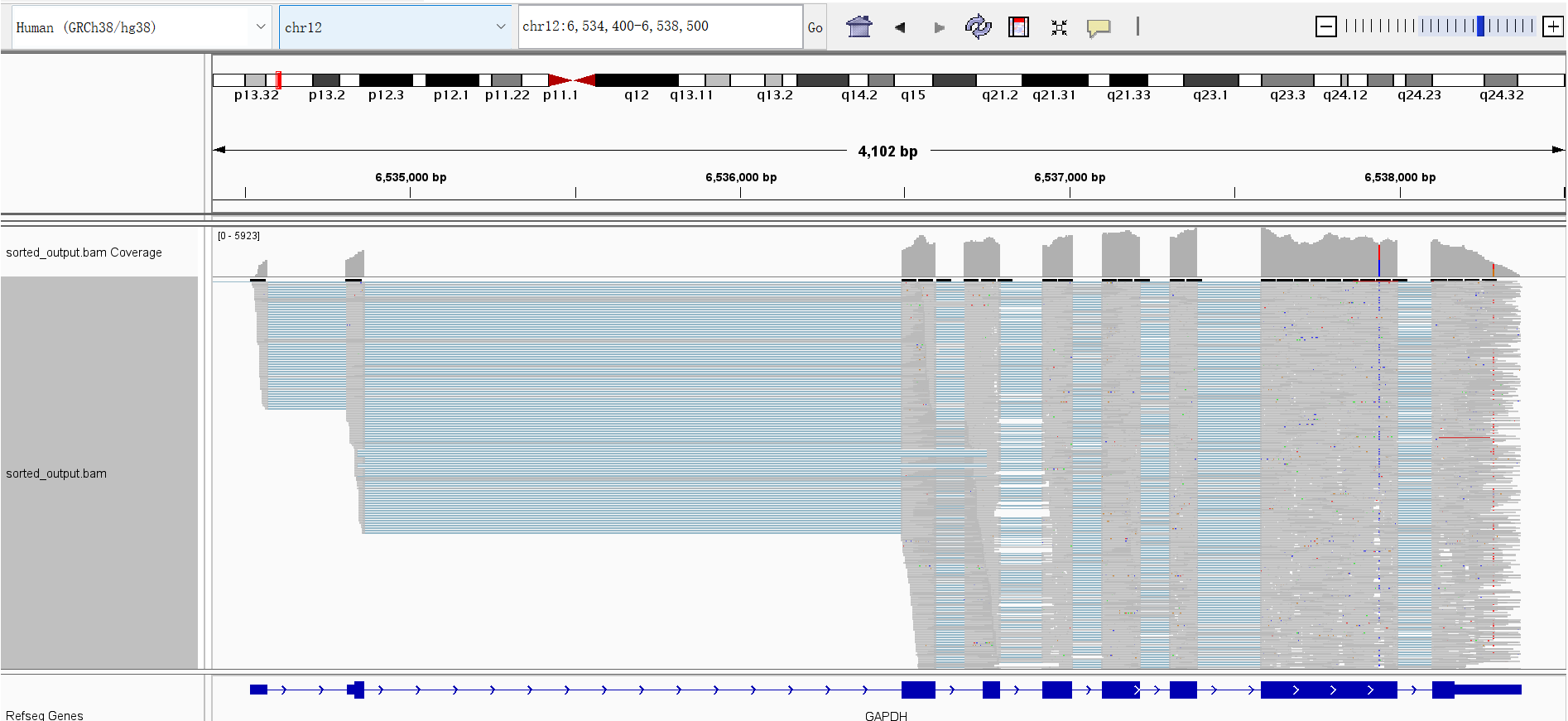


**Supplemental Fig. S10**. Visualization of sequencing depth across nine exons in the GAPDH-201 transcript using the Integrative Genomics Viewer (IGV). This figure highlights the variation in read coverage for paired-end fragments along the genomic reference. Base-calling errors are also indicated dispersed across the mapped reads.

Fig. S11.


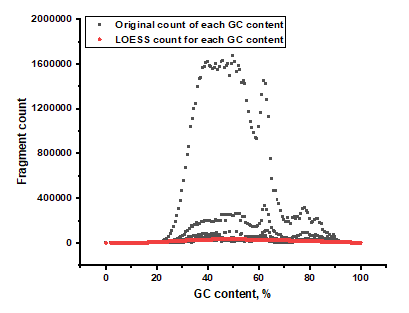


**Supplemental Fig. S11**. GC bias correction methodology employs LOESS regression to fit a relationship between the GC content of un-binned fragments and their sequencing counts across transcripts, based on empirical data. This relationship helps adjust for biases in sequencing data attributable to GC content variation.

Fig. S12.


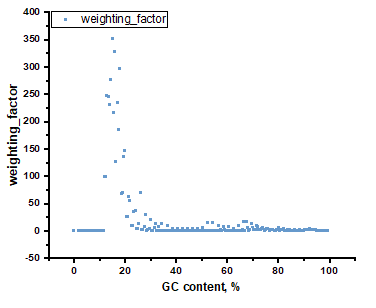


**Supplemental Fig. S12**. This figure depicts the weighting factors assigned to each 0.5-interval binned GC content across the entire transcriptome, as determined by LOESS regression analysis.

Fig. S13.


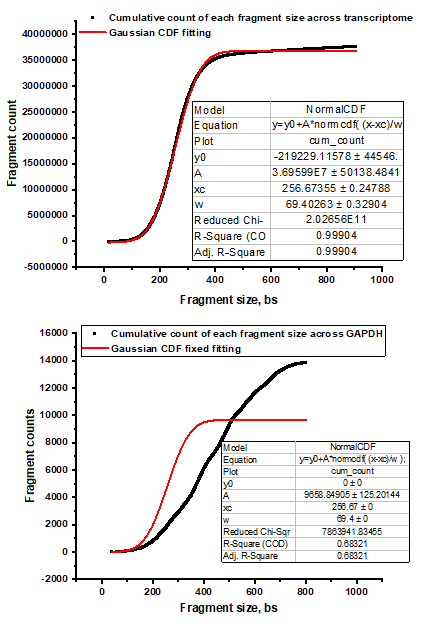


**Supplemental Fig. S13**. The cumulative fragment counts for all fragments across the entire transcriptome are modeled using an empirical cumulative distribution function (CDF). The key parameters for this function have been predetermined and are displayed in the upper panel of the analysis. For the GAPDH transcript specifically, its cumulative fragment count is also fitted using this parameter-fixed CDF. The amplitude, representing the cumulative fragment count specific to the GAPDH transcript, is then determined and presented as the calibrated abundance in the lower panel of the analysis.

Fig. S14.


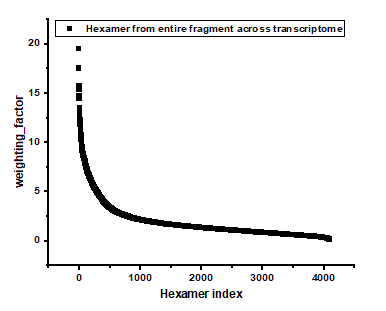


**Supplemental Fig. S14**. Calculation of weighting factors for each hexamer sequence from a total of 4096 (4^6^) possible sequences. Hexamer counts are derived from the initial six bases of both ends of paired-end fragments spanning the entire transcriptome.

Fig. S15.


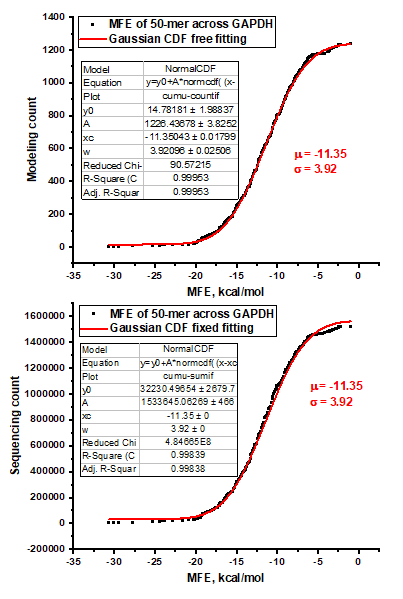


**Supplemental Fig. S15**. Analysis of the cumulative count of 50-mer sequences across the GAPDH transcript using a Gaussian Cumulative Distribution Function (CDF). This graph displays an even distribution of all 50-mers, where key parameters for the function were predetermined. The sequencing data's cumulative 50-mer count was also aligned using the fixed-parameter CDF. The amplitude, indicative of the cumulative 50-mer count, is detailed and presented as the calibrated k-mer abundance in the lower panel of the analysis.

Fig. S16.


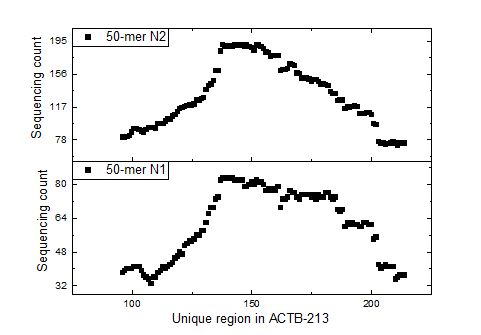


**Supplemental Fig. S16**. The profile of sequencing counts in the unique region corresponding to the ACTB-213 transcript reference across replicated samples.

Fig. S17.


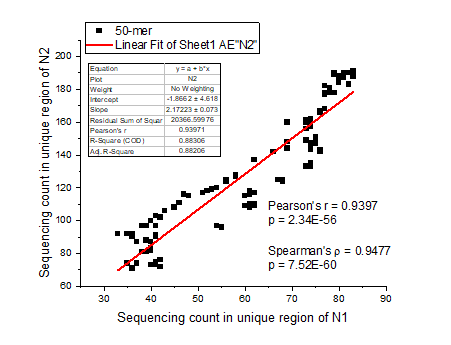


**Supplemental Fig. S17**. Linear regression was conducted on the sequencing counts of 50-mer sequences across unique segments of the ACTB0213 transcript template. The analysis included calculating the Pearson and Spearman correlation coefficients, with p-values assessed to evaluate the statistical significance.

Fig. S18.


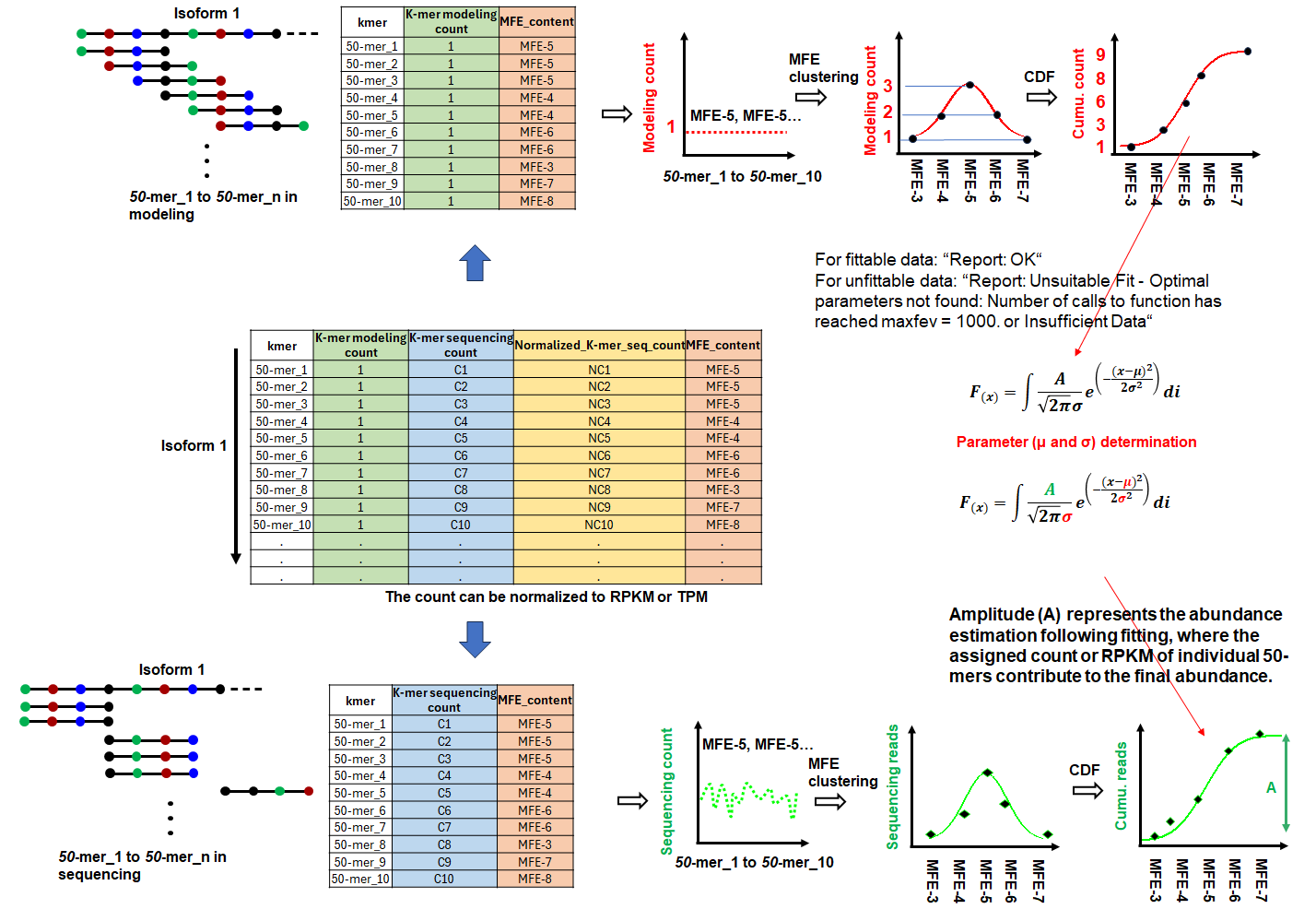


**Supplemental Fig. S18**. Transcript-specific benchmarking parameters determination. The transcript-specific benchmarking parameters are determined by categorizing evenly-distributed 50-mers throughout the transcript based on their Minimum Free Energy (MFE) content and integrating individual occurrence counts within each MFE category. The table presents data acquisition from the sequence data and transcript sequence, focusing on the columns: k-mer modeling count and MFE content. To derive key parameters, the resultant MFE content-enumerated counts are fit to a pseudo-Gaussian distribution function, and an accumulative distribution function (CDF) is applied to accommodate the cumulative MFE content-related counts. This process yields the two fundamental parameters: mean (μ) and standard deviation (σ). Uneven k-mer coverage, exhibiting position-specific occurrence counting facets of the sequencing data, forms the columns of k-mer sequencing count (or normalized k-mer sequencing count) and associated MFE content. The k-mers, categorized by MFE content, are summed within each category and plotted. Once the parameters derived from the modeling data are set, the pseudo-Gaussian distribution function aptly acknowledges all intrinsic biases in the sequencing data. Additionally, by applying the pseudo-CDF with the fixed parameters, accurate estimates of transcript abundance are achieved by considering the cumulative effect on k-mer occurrence count.

Fig. S19.


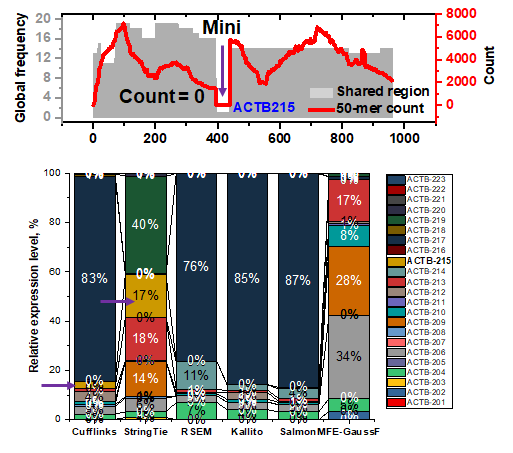


Figure 19. Up) Isoform-level transcript quantification of ACTB using the MFE-GSB-based model. This section highlights the profiling and parametric quantification of the ACTB-215 isoform (arrow). A detailed examination of this isoform is performed using 50-mer sequencing counts for transcript abundance profiling. Down) Stacked bar chart illustrating the abundance proportions of the 23 ACTB isoforms, as determined by various transcript quantification methods, including Kallisto, Salmon, Cufflinks, StringTie, RSEM, and MFE-GSB-MFE-GaussF. Each bar in the chart represents the contribution of individual isoforms to the total mapped reads for each method. Of particular interest, marked with an arrow, is the ACTB-215 isoform. This visualization provides insight into how each quantification method distributes read contributions across the various ACTB isoforms.
